# Recent Advances on Two-Dimensional Nanomaterials Supported Single-Atom for Hydrogen Evolution Electrocatalysts

**DOI:** 10.3390/molecules29184304

**Published:** 2024-09-11

**Authors:** Kangkai Fu, Douke Yuan, Ting Yu, Chaojun Lei, Zhenhui Kou, Bingfeng Huang, Siliu Lyu, Feng Zhang, Tongtao Wan

**Affiliations:** 1 Hubei Key Laboratory of Automotive Power Train and Electronic Control, School of Automotive Engineering, Hubei University of Automotive Technology, Shiyan 442002, China; 202204448@huat.edu.cn (K.F.); 202204437@huat.edu.cn (D.Y.); yuting@huat.edu.cn (T.Y.); huangbf_qc@huat.edu.cn (B.H.); 2 Hubei Key Laboratory of Energy Storage and Power Battery, School of Mathematics, Physics and Optoelectronic Engineering, Hubei University of Automotive Technology, Shiyan 442002, China; 3 Key Laboratory of Organosilicon Chemistry and Material Technology, College of Material, Chemistry and Chemical Engineering, Ministry of Education, Hangzhou Normal University, Hangzhou 311121, China; chaojun_lei@hznu.edu.cn; 4 Key Laboratory of Biomass Chemical Engineering of Ministry of Education, College of Chemical and Biological Engineering, Zhejiang University, Hangzhou 310027, China; 12328078@zju.edu.cn

**Keywords:** two-dimensional materials, single-atom catalysts, electrocatalytic, hydrogen evolution reaction, metal–support interactions

## Abstract

Water electrolysis has been recognized as a promising technology that can convert renewable energy into hydrogen for storage and utilization. The superior activity and low cost of catalysis are key factors in promoting the industrialization of water electrolysis. Single-atom catalysts (SACs) have attracted attention due to their ultra-high atomic utilization, clear structure, and highest hydrogen evolution reaction (HER) performance. In addition, the performance and stability of single-atom (SA) substrates are crucial, and various two-dimensional (2D) nanomaterial supports have become promising foundations for SA due to their unique exposed surfaces, diverse elemental compositions, and flexible electronic structures, to drive single atoms to reach performance limits. The SA supported by 2D nanomaterials exhibits various electronic interactions and synergistic effects, all of which need to be comprehensively summarized. This article aims to organize and discuss the progress of 2D nanomaterial single-atom supports in enhancing HER, including common and widely used synthesis methods, advanced characterization techniques, different types of 2D supports, and the correlation between structural hydrogen evolution performance. Finally, the latest understanding of 2D nanomaterial supports was proposed.

## 1. Introduction

Clean and efficient renewable energy sources are essential for reducing carbon dioxide emissions and promoting sustainable development [1,2,3,4]. While solar, wind, geothermal, and biomass energy technologies have advanced significantly, the limitations hinder widespread implementation [5,6,7]. This has sparked interest in clean gaseous energy sources, particularly hydrogen, which is known for its zero-emission combustion and potential to drive innovation in emerging energy technologies [8,9,10,11,12,13,14,15]. HER is considered one of the most essential clean energy technologies for sustainable development, and HER is also a vital model reaction for designing other novel electrocatalysts and understanding electrochemical reactions [16]. HER refers to when electrolyzing water, protons, or hydrated hydrogen ions obtain electrons in the cathode and conduct a reduction reaction to generate hydrogen. Cathodic hydrogen evolution can occur based on the Volmer–Heyrovsky mechanism or the Volmer–Tafel mechanism.

In acidic electrolytes:

H^+^ + e^−^ + * → H* (Volmer reaction)

H_ads_ + H^+^ + e^-^ → H_2_ (Heyrovsky reaction)

H_ads_ + H_ads_ → H_2_ (Tafel reaction)

In alkaline electrolytes:

H_2_O + e^−^ + * → H_ads_ + OH^−^ (Volmer reaction)

H_ads_ + H_2_O + e^−^ → H_2_ + OH^−^ (Heyrovsky reaction)

H_ads_ + H_ads_ → H_2_ (Tafel reaction)

where * represents the active sites on the catalyst surface, and ads represents the adsorption state of intermediates (H_ads_). However, implementing efficient water decomposition techniques is a huge challenge due to the kinetic barriers of HER, so the efficiency of HER mainly depends on the activity of the electrocatalyst. The efficiency of HER is affected by potential, Tafel slope, stability, and electrolyte.

Overpotential refers to the part in which the actual voltage exceeds the theoretical voltage when a certain current density is reached in the electrocatalytic reaction. Ideally, the operating potential required for an electrocatalytic reaction is the potential at equilibrium. However, the working potential in the actual reaction often needs to overcome the obstacles of the kinetic process to exhibit a value higher than the equilibrium potential. Overpotential is mainly used to overcome other resistors such as activation resistance and charge transfer resistance; theoretically speaking, the closer the overpotential η is to 0 V, the better the performance of the catalyst [17]. The basic formula of overpotential can be expressed as:

η = E_i_ − E_t_

η: overpotential E_i_: actual potential E_t_: theoretical potential

The Tafel slope provides an important reference for exploring the reaction mechanism, especially in clarifying the reaction rate-determining steps and reaction paths, such as HER reaction step 1 (H+ + e^−^ + * → H_ads_) in acidic conditions and HER reaction step 1 (H_2_O + e^−^ → OH^−^ + H_ads_) in alkaline conditions mentioned above. During the HER, the Tafel slope refers to the slope of the relationship between current density and electrode potential in the kinetic process of the HER electrode. The low value of the Tafel slope presents high-speed increases in current density increases, indicating fast catalyst kinetics and good catalytic activity [18].

Stability is an extremely important evaluation index. The catalysts that can be used for practical applications are the stability of the performance, in order to prove the stability of the catalysts, it is more common to use chronopotentiometry and concurrent methods. Chronopotentiometry sets a constant current density and records the change in potential over time. The ignored potential change with time indicates good stability performance of catalysts. Chronoamperometry is to set a certain overpotential and record the change in overpotential with time. The ignored current density change with time presents good stability of catalysts [19].

Electrolytes are an important part of electrochemical reactions because electrolytes affect the environment of chemical reactions. According to the different electrolytes, these can be divided into alkaline solution, acidic solution, and proton exchange membrane. Electrolyte ions include halogen anions, carbonate anions, and alkali metal cations. The electrolytes are not only a medium for transporting electrons, the electrolyte ions have many complex mechanisms, including chemical interactions, electrostatic interactions, interfacial pH buffering, and changes in the adsorption structure of the reactants [19].

Research on catalyst design, synthesis, and catalytic mechanisms has progressed from the nanoscale and microscale to the atomic and subatomic levels [20,21]. Single-atom catalysts (SACs) consist of a single atom (SA) on the support and the substrate, exhibit exceptional catalytic performance, and have been extensively researched [22]. Two-dimensional nanomaterials supported by SAs and clusters have become a new frontier of energy catalysis due to their excellent catalytic performances and good recyclability [23]. However, clusters still have some defects compared to SAs. The catalytic mass activity and stability of atomic clusters are not satisfactory due to the unfavorable hydrolysis dissociation and high surface energy in the Volmer step, which leads to a decrease in atom utilization [24]. Unlike atomic clusters, the simple structure of the single atom enables the SACs to have a clear active site, which can optimize the reaction path through defect engineering or interaction [25]. In addition, differing from the traditional clusters, the unique electronic structures and energy level orbitals of SACs can maximize the atomic utilization of single atoms. Moreover, the SACs present a more accessible microenvironment of active sites and an easier comprehensive understanding of HER mechanisms [26].

Single-atom catalysts can be divided into noble metal catalysts and transition metal catalysts in simple terms. Noble metal catalysts refer to catalysts containing noble metals (Pt, Ru, Ir, etc.). Noble metal catalysts have been widely discussed by researchers because of their high selectivity, high activity, maximum atomic utilization, and unique electronic structure. However, the scarce resources and high prices of noble metal catalysts are not suitable for large-scale practical applications [27].

Transition metal catalysts include transition metal hydroxides, oxides, sulfides, phosphates, alloys, and so on. Transition metal catalysts have abundant reserves, low cost, good stability, diverse element composition, controllable lattice structure, flexible electronic structure, rich active sites, and can improve selectivity through metal–carrier interface [27]. However, due to the simple synthesis conditions of some transition metal catalysts, it is difficult to accurately disperse metal sites at the atomic level under high temperatures and pressure [28]. These characteristics make SACs promising for hydrogen energy utilization, positioning them as a key focus for future research. The supports of single atoms are crucial for SACs, which play a vital role in anchoring individual atoms and controlling catalyst performance and stability [29,30]. Recently, various carbon materials like graphene and carbon nitrides have been used as supports for SAs, along with non-carbon materials such as TiO_2_ and CeO_2_ [31,32]. Two-dimensional materials, in particular, offer unique advantages as supports due to the well-defined surfaces that expose numerous active sites [32].

Two-dimensional materials can comprise a range of elements, display diverse crystal structures, and exhibit malleable, adaptable electronic properties. The homogeneous surfaces of 2D supports enable uniform surface modifications, making them highly promising platforms for loading and modifying SAs. Two-dimensional materials have a higher specific surface area than three-dimensional materials, which allows single atoms to be more evenly dispersed across the surfaces. This distribution increases the load efficiency of individual atoms. When used as a support for SACs, this property enables SACs to significantly increase catalytic activity [33]. Two-dimensional materials have more variable structures and better ductility, which can be modified by different anchoring methods and interactions to increase the active site [34]. In addition, 2D materials have simpler chemical synthesis methods, and abundant 2D materials can help to fully understand the reaction mechanism and catalytic activity of 2D materials in the field of electrocatalysis [35]. Consequently, 2D materials have emerged as one of the most promising supports for loading and modifying SAs [36,37]. Abundant defects and specific lattice substitutional atoms on the surface of 2D carbon materials tend to bond with SAs [38], the phenomenon plays a role in regulating the rich electron states in the d-orbitals of the active metal atoms and positively affects the charge transfer between the SAs and the supports [39]. In studies involving 2D non-carbon support, the construction of diverse electronic structures on the surface leads to various interactions between SAs and the 2D supports [40,41,42], including surface defects [43,44], specific atomic doping [45], and organic functional group binding [46]. In terms of catalyst stability, the strong interactions between 2D supports and SAs are crucial for anchoring SAs and preventing the aggregation of SAs [47]. Additionally, the rich forms of SAs on the support surface, such as protrusions [48,49], doping [50,51], and vacancies [52,53], greatly diversify the types of SACs. Some lattice atoms on the surface of 2D supports are thought to play an auxiliary role in catalytic reactions, contributing to a synergistic effect in reactions like the HER [54]. Therefore, the study of the structure–property interactions between well-defined SA sites and structurally clear 2D supports is an ideal platform for investigating the mechanisms underlying the performance of SACs [55,56]. Two-dimensional materials have become a popular choice for supporting and modifying SAs [57,58,59].

This review provides a comprehensive review of the research progress on 2D support materials, emphasizing the distinct advantages of using 2D materials as support for SAs. The various anchoring methods and interaction modes of SAs on complex 2D support surfaces necessitate elevated standards for material synthesis and characterization techniques. The advancements of new synthesis technologies and the applications of characterization methods are crucial in uncovering the origins of SAC activity. The integration of diverse high-resolution instruments and progress in in situ characterization techniques bring researchers closer to comprehending performance mechanisms. Furthermore, the article delves into the current state of SACs loaded on 2D support materials for HER reactions, exploring multiple interaction modes to gain a deeper insight into the unique roles of SA sites on the surfaces of 2D support materials. The introduction of various performance optimization methods and the proposal of intrinsic mechanisms underscore the advantages of utilizing 2D materials loaded with SACs. This research establishes the theoretical groundwork for the practical application of SACs. In the outlook section, the article briefly addresses the remaining challenges in both the mechanisms and applications of SACs.

## 2. Synthetic Approaches and Characterization Methods

In recent years, the field of electrocatalysis has witnessed significant advances with the advent of 2D materials loaded with SAs. The electrocatalytic performance of 2D materials loaded with SAs is influenced by their material composition, element content, thickness, shape, surface properties, and interactions with SAs. The ability to prepare these high-performance loaded single-atom 2D materials requires reliable preparation methods and advanced precision characterization instruments. In this section, the widely used preparation methods and characterization techniques are mainly introduced in recent years. The preparation methods are explained in two parts: “2D materials” and “loaded SAs”, and characterization techniques are introduced from three aspects: spectroscopy, imaging, and simulation calculation. In addition, the catalytic mechanism and correlation between SAs and 2D materials are explored through characterization techniques.

### 2.1. Synthetic Approaches of 2D Single-Atom Supports

This section introduces the preparation methods of 2D materials from two aspects: the “top-down” method and the “bottom-up” method. The “top-down” method mainly includes micromechanical peeling, mechanical force-assisted liquid phase stripping, ion insertion-assisted liquid phase exfoliation, ion exchange-assisted liquid phase exfoliation, oxidation-assisted liquid phase exfoliation, and selective etching-assisted liquid phase exfoliation. The “bottom-up” method mainly includes chemical vapor deposition and wet chemical methods (hydrothermal synthesis, nanocrystalline self-assembly, 2D template synthesis method, heat injection method, and interface mediated synthesis method). These methods vary in terms of operational difficulty, cost, applicability, and safety. The following will mainly introduce the operating principles, advantages and disadvantages, and suitability for preparing 2D materials of these methods.

#### 2.1.1. “Top-Down” Method

*Micromechanical Peeling.* The micromechanical peeling method involves using transparent adhesive tape to separate layers of bulk crystals by breaking the weak van der Waals force between them while keeping the covalent bonds within each layer intact. Geim et al. first applied this method in 2004 to successfully exfoliate single-layer graphene from highly oriented pyrolytic graphite surfaces [60]. The advantage of this method is that it does not require the addition of other materials for reaction, resulting in a high content of 2D materials. However, repeated use of transparent tape peeling makes the preparation efficiency of 2D materials too low, and due to the uncontrollability of the tape, the size and shape of the prepared 2D materials cannot be controlled. After continuous optimization, this method is widely used for the preparation of 2D materials such as MoSe_2_, WS_2_, WSe_2_, MoTe_2_, ReS_2_, MoS_2_, etc.

*Mechanical force-assisted liquid phase stripping.* The mechanical force-assisted liquid phase stripping method involves the application of mechanical forces (such as acoustic waves and shear) in a liquid to disrupt the van der Waals forces between layers, resulting in the production of 2D nanosheets. Mechanical force-assisted liquid phase stripping can be divided into acoustic wave-assisted liquid phase stripping and shear force-assisted liquid phase stripping. The acoustic-assisted liquid-phase exfoliation method involves dispersing bulk materials into a solvent for acoustic degradation. A large number of cavitation bubbles are distributed around graphite, and when the bubbles rupture, shock waves and microjets are generated. When the shock waves are transmitted to the free interface of graphite, tensile stress waves are reflected, and countless cavitation bubbles rupture, generating strong tensile stress around graphite for exfoliation (Figure 1a) [61]. Shaijumon et al. synthesized MoS_2_ quantum dots with a size of 2 nm using ultrasound-assisted liquid-phase exfoliation in 2014 (Figure 1b,c), resulting in a higher concentration of MoS_2_ active edge sites and stronger HER activity [61]. The method is simple and cost-effective, but the peeling efficiency is low, the size of the nanosheets is small, and impurities are easily introduced. In addition to being able to exfoliate graphene, this method has also been applied to prepare various 2D materials such as TMDs (MoS_2_, WS_2_, MoSe_2_, MoTe_2_, TaSe_2_, NbSe_2_, NiTe_2_, and Bi_2_Te_3_), h-BN, and TMOs (MoO_3_, WO_3_, etc.). Different from the above method, the shear-assisted liquid-phase exfoliation method utilizes the shear force generated by the rotor stator stirrer for exfoliation. Coleman et al. first proposed using a rotor stator stirrer to exfoliate graphene from graphite in 2014 and later used this method to exfoliate h-BN, MoS_2_, and WS_2_ [62]. The shear-assisted liquid-phase exfoliation method has high exfoliation efficiency and is a promising exfoliation technique. Currently, this method has been widely used in the commercial production of graphene.

*Ion insertion-assisted liquid phase exfoliation.* The method involves introducing small-radius cations into the layers of bulk material to form an intercalation compound. This process causes the crystals to expand and weakens the van der Waals forces between the layers, making them more easily separable through mechanical means. According to the different ways of ion intercalation, it can be divided into chemical intercalation and electrochemical intercalation, using organic metal compounds and metal foils as ion sources, respectively. Graphene, h-BN, and numerous TMDs can be prepared. Lin et al. utilize this technique to produce high-quality 2D layered crystalline materials such as MoS_2_, MoSe_2_, MoTe_2_, WS_2_, HfS_2_, NbSe_2_, Bi_2_Se_3_, Sb_2_Te_3_, In_2_Se_3_, InSe, black phosphorus, and other similar materials by inserting organic molecules between the layers [63]. The advantage of this method is high yield, but the degree of ion insertion in chemical intercalation is difficult to control and requires a rigorous experimental environment. The electrochemical intercalation process is complex and prone to introducing impurities.

*Ion exchange-assisted liquid phase exfoliation.* This method employs the use of larger organic or inorganic ions to replace the smaller ions originally located between the layers of a material. This results in an increase in the interlayer spacing, thereby facilitating the separation of the layers through mechanical forces [64]. This method is mainly divided into cation exchange assisted and anion exchange assisted. Cation exchange can prepare layered metal oxides and metal phosphorus trisulfides, while anion exchange can prepare layered double hydroxides (LDHs) nanosheets. Song et al. introduced dispersed NiFe LDH into formamide using the anion exchange-assisted liquid-phase exfoliation method and prepared monolayer LDHs by stirring [65]. The advantage of this method lies in the potential for efficient and large-scale production of specific 2D materials, but there are chemical reactions and deviations in product com*Oxidation-assisted liquid phase exfoliation.* This process involves the use of strong oxidants to convert graphite into graphene oxide (GO). The oxidation introduces a multitude of functional groups onto the graphene’s surface, which increases the interlayer spacing and facilitates the separation of the layers [66]. Yang et al. utilize oxidation-assisted liquid-phase exfoliation and microwave treatment to prepare expanded graphite by reacting graphite with strong oxidants K_2_S_2_O_8_ and H_2_SO_4_ [67]. The advantage of this method is that it can efficiently produce graphene, but the limitations are too great to prepare other 2D materials, and the preparation risk increases with the increase in strong oxidants.

*Selective etching-assisted liquid phase exfoliation.* The process involves using an etchant, such as hydrofluoric acid or a fluoride, to selectively remove the atomic layer from MAX phase materials. Here, in MAX phase materials, M refers to metallic elements, A refers to the main group elements, and X refers to carbon, nitrogen, or boron. This allows for the separation of 2D MXenes using acoustic waves [68]. Gogotsi et al. demonstrate the preparation of 2D titanium nitride Ti_4_N_3_ through a selective etching-assisted liquid-phase exfoliation method. The Al is etched from Ti_4_AlN_3_ powder precursors by utilizing molten fluoride salts in an argon atmosphere at 550 °C. Subsequently, the obtained MXenes are layered to generate few-layered nanosheets and Ti_4_N_3_T_x_ monolayers, where T represents the surface termination (F, O, or OH) [69]. The advantage of this method is that it is very effective in preparing MXenes and is often used to prepare more such materials. However, it has significant limitations and is difficult to promote in the preparation of other materials. Moreover, corrosive etchants may pose experimental risks. 

#### 2.1.2. “Bottom Up” Approach

**Figure 2:**
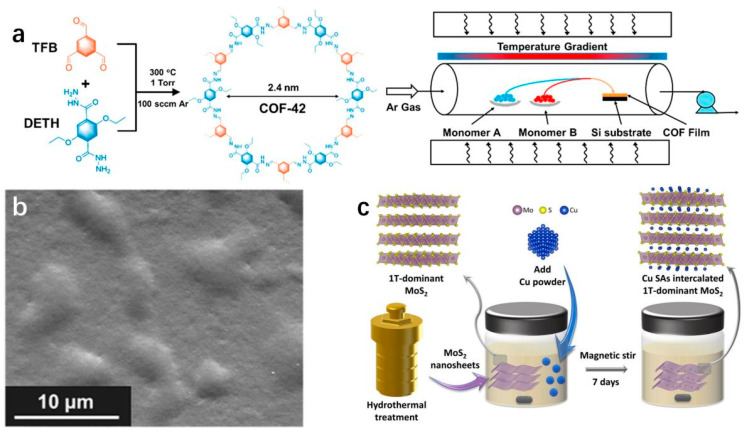
(**a**) Gas phase reaction chemical process for the production of dihydrazine COF-42 films [70]. (**b**) SEM image of the COF-42 film [70]. Copyright 2023, American Chemical Society. (**c**) Schematic diagram of preparation of Cu_x_MoS_2_ by hydrothermal method [72]. Copyright 2023, Wiley-VCH.

*Chemical vapor deposition (CVD).* CVD mainly involves circulating one or several gas-phase precursors into a reaction furnace, where they react or decompose on the substrate surface at high temperatures to obtain 2D materials such as graphene and transition metal carbides, ensuring high crystallinity, purity, and yield of the 2D materials. The advantages of this method lie in the controllability of material size, thickness, and composition, but the production cost is relatively high. Verduzco et al. utilize CVD to produce a covalent organic framework (COF) film called COF-42 that is structurally sound and defect-free (Figure 2a,b) [70].

*Wet chemical synthesis.* The method used to produce non-layered structured materials in the realm of 2D materials, involves reactions of precursors in a liquid phase. Various types of wet chemical synthesis techniques include hydrothermal synthesis, nanocrystalline self-assembly, 2D template synthesis, thermal injection, interface-mediated synthesis, and surface synthesis [71].

*Hydrothermal synthesis.* The use of water or another solvent as the reaction medium within a sealed environment is known as hydrothermal synthesis. This method is characterized by its high efficiency and cost-effectiveness, although it provides limited control over the process. Yi et al. prepare 1T phase MoS_2_ and Cu-SAs using the hydrothermal method, providing samples for the subsequent preparation of Cu_0.39_MoS_2_ (Figure 2c) [72].

*Nanocrystalline self-assembly*. The method involves organizing nanoparticles or nanowires into 2D crystal structures using non-covalent interactions, leading to the creation of monolayer nanomaterials with precise morphologies. Yang et al. demonstrate the successful synthesis of a 2D FeP nanoframework superlattice from carbon-coated Fe_3_O_4_ nanocube superlattices, achieved through the Kirkendall effect and a spatially confined chemical transformation process (Figure 3a). Among them, NCSLs refer to nanocube superlattices [73].

*Two-dimensional template synthesis method.* The 2D template synthesis approach utilizes pre-existing nanomaterials or support to guide the growth of specific nanostructures into unique forms with distinct properties, such as high anisotropy or hollow structures. Zhu et al. demonstrate the effectiveness of this method by using amorphous metal oxide nanosheets as templates to produce a significant amount of ultra-thin 2D metal–organic frameworks (MOF) nanosheets through the combination of various ligands (Figure 3b) [74].

*Heat injection method.* The method involves rapidly introducing highly reactive substances into a heated surfactant solution, leading to the formation of uniform nanocrystals that are pure and consistent in size and shape. Li et al. utilize this method to synthesize uniform and pure nanocrystals by rapidly introducing reactive substances into a heated surfactant solution (Figure 4a) [75].

*Interface-mediated synthesis method.* The synthesis of metal-coordinated polymers is primarily achieved through interface-mediated processes. This approach involves confining organic ligands at interfaces such as water/air or organic solvent/air, which can react with metal salts to produce 2D materials. Kan et al. investigate the synthesis of 2D N-graphene films at gas/liquid and liquid/liquid interfaces by polymerizing triazine or pyrazine-based monomers with acetylene groups through a Glazer coupling reaction at the interface (Figure 4b) [76].

Overall, the “top-down” method is more suitable for preparing 2D materials by peeling materials with three-dimensional layered structures. The “bottom-up” method is more suitable for chemical reactions of precursors under specific conditions, both of which play a very important role in the field of 2D materials, providing a bright and broad path for the preparation of 2D materials. However, there is still potential for development in the preparation process.

### 2.2. Synthetic Strategies of 2D-TMD-Supported SACs

The advancement of SACs and 2D support materials has led to rapid progress in loading methods for SA sites. These different loading techniques affect the efficiency and configurations of SAs, as well as the interaction modes between SAs and 2D support. However, to fabricate theoretically designed SACs, it is still difficult to manipulate the atoms in a highly precise way. Driven by the ultra-high surface free energy, individual atoms easily migrate and aggregate into nanoparticles. Therefore, to reduce the free energy of the system or enhance the energy barrier of atomic agglomeration, a strong SA-2D support interaction must be formed. Consequently, the development of various loading methods for SAs plays a crucial role in the research of SACs on 2D support.

**Figure 5:**
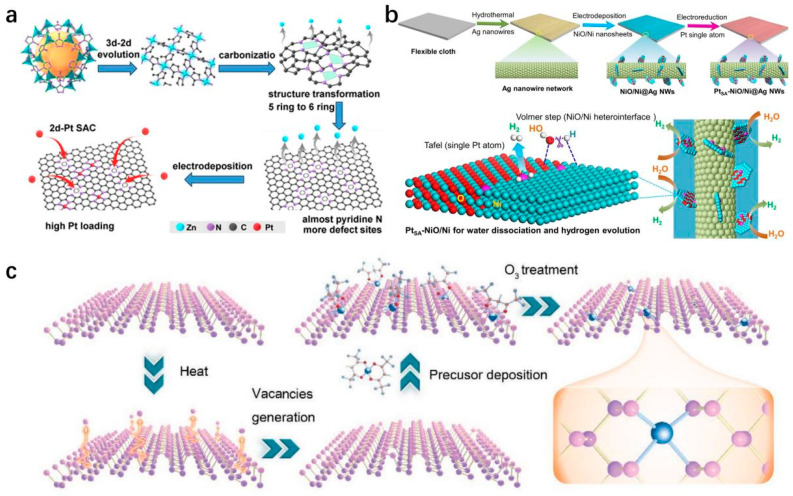
(**a**) 3d-2d evolution mechanism and corresponding carbonization process diagram [78]. Copyright 2019, Wiley-VCH. (**b**) Pt-SA anchored NiO/Ni heterostructure nanosheets on Ag nanowires network [80]. Copyright 2021, Springer Nature. (**c**) BP confined SACs via ALD [81]. Copyright 2021, Wiley-VCH.

*Electrochemical Deposition.* SACs can be synthesized easily using electrodeposition methods, where metal ions dissolved in an electrolyte solution are loaded onto a support material. The density of the distribution of metal monoatoms can be precisely controlled by adjusting the concentration of metal ions and the deposition time [77]. For example, in the case of depositing Pt-SA on a support, a Pt foil is used as the source of the metal precursor. By applying a high oxidation potential to the Pt counter electrode in a three-electrode system, the Pt foil is oxidized and dissolved in the electrolyte. As shown in Figure 5a [78], Zhang et al. utilize a three-electrode system to electrodeposit Pt-SA on a 2D layered support, setting the electrodeposition potential range between −0.35 V and −0.20 V concerning the reversible hydrogen electrode (RHE) to facilitate the HER. The deposited Pt atoms show a promoting effect on the HER, leading to a gradual enhancement in current density during the deposition process. This enhancement in current density can be considered as an indication of the successful deposition of Pt atoms [78].

*Wet Chemistry.* The method is considered the primary approach for SAC synthesis because of its straightforwardness and ability to be scaled up. Choosing the appropriate catalyst supports and carefully adjusting the concentration of metal ion precursors are key factors in controlling metal–support interactions, which are vital for maintaining SAC stability. Strong metal–support interactions are necessary to prevent aggregation and facilitate the formation of SACs [79].

*Electrochemical Reduction.* The method used to deposit SAs onto ditopic materials by controlling chemical reaction conditions and surface energy. This technique enables the creation of nanomaterials with specific functions and properties. As described in Figure 5b [80], Zhou et al. initially fabricated Ag NWs on flexible support through a chemical process, followed by the generation of Ni/NiO nanosheets on the Ag NWs using a straightforward approach. Subsequently, Pt_SA_-NiO/Ni is synthesized by reducing Pt ions in solution using the electrochemical reduction method in an alkaline solution with a low concentration of H_2_PtCl_6_. The NiO/Ni heterostructure exhibits numerous holes and O vacancies resulting from crystal defects, which serve as sites for anchoring Pt-SA [80].

*Atomic Layer Deposition (ALD)*. ALD provides a precise and controlled approach for synthesizing SACs by means of self-limiting surface reactions. Chen et al. effectively load individual metal atoms onto 2D black phosphorus (BP) using ALD. Prior to introducing the metal precursor molecules, the exfoliated BP sheets are heated to 150 °C in the ALD chamber to eliminate any potential surface adsorbents. This heating process within the specified temperature range also promoted the generation of additional vacancies on the surface, which acted as anchoring sites. Subsequently, metal–organic precursors are deposited and anchored in these vacant sites on the BP flakes, forming metal–phosphorus bonds by partially eliminating the organic ligands. Further treatment with ozone removed any remaining ligands from the deposited metal precursors, revealing the anchored SAs on the BP surface (Figure 5c) [81]. The precise loading of isolated metal atoms could be regulated by adjusting the exposure time to the precursor. Once all anchoring sites were fully utilized, the maximum loading of the SACs could be attained [81].

*The Ion Exchange Method.* The method involves the transfer of metal ions onto the surface of support, resulting in the formation of a single-layer catalyst. The selection of appropriate supports is crucial to maximize ion exchange capacity. Metal ions are first dissolved in a solvent and then react with the support to create the catalyst. This method offers precise control over the catalyst’s composition and structure [82]. As seen in Figure 6a,b [83]. Sun et al. successfully fabricated pristine MOF nanosheets on Ni foam using a hydrothermal approach. The original MOF material, known as “Ni-BDC”, served as the foundation for synthesizing Ru-SA catalysts by replacing some Ni atoms. The resulting NiRu_0.13_-BDC catalyst demonstrates excellent activity across various pH levels, especially in a 1 M phosphate-buffered salt solution at current densities of up to 10 mA cm^−2^. Furthermore, it displays a low overpotential of 36 mV, similar to that of commercial Pt/C catalysts [83].

*Heat Treatment.* Synthesize Pt-SA onto Ti_3_C_2_T_x_ by heating it in a hydrogen atmosphere. As shown in Figure 6c [84], the oxygen vacancies (O_V_) on monolayers of Ti_3_C_2_T_x_ in a reducing atmosphere can trap Pt-SA through the formation of Pt-Ti bonds. The anchoring of Pt-SAs to O_V_ on Ti_3_C_2_T_x_ leads to a reduction in the binding energy for hydrogen adsorption and the strength of hybridization with hydrogen, thereby demonstrating superior catalytic activity [84].

### 2.3. Characterization Methods

Advanced high-resolution characterization instruments provide a valuable means for identifying SA sites and understanding the electronic interactions between SAs and support. This aids in studying the catalytic mechanism of SACs and reveals the correlation between catalyst structure and performance [85]. Currently, the structural characterization methods of SACs can be categorized into two groups: direct imaging and indirect spectroscopy. High-resolution imaging allows researchers to visually observe the distribution of SAs on the support surface, including their loading form, specific position, and distance from other atoms. Spectroscopic techniques provide detailed information on the local coordination structure of SAs, such as coordination number and bond length, offering an objective measure for structure–property relationships [86]. These techniques can track dynamic changes on the catalyst surface during reactions, including catalyst performance reconstruction and adsorption properties of reaction intermediates [87]. In addition, DFT, as an important part of the optimization and design of single-atom theoretical models, can help researchers predict promising single-atom catalysts [88]. This section will provide a comprehensive overview of the various SA characterization techniques used to investigate interactions between SAs and support.

#### 2.3.1. Imaging Characterization Techniques

**Figure 7:**
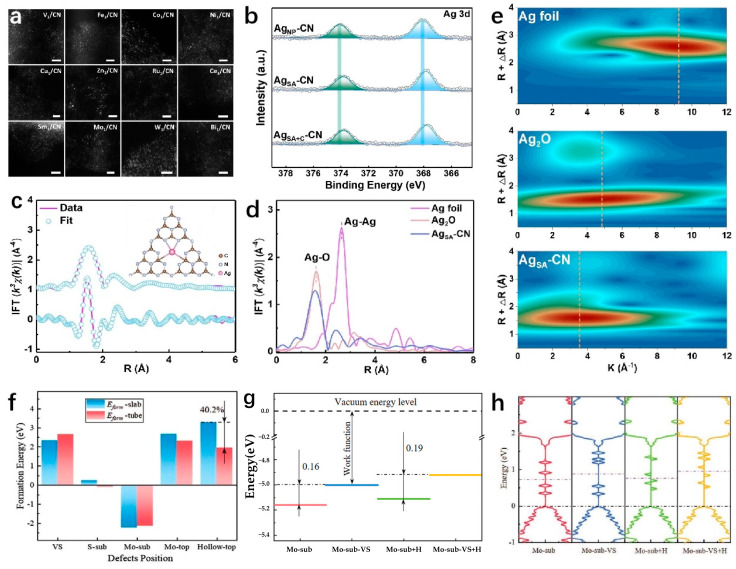
(**a**) AC-HAADF-STEM images of SACs prepared using different metal atoms. Scale bars: 2 nm [90]. Copyright 2021, Elsevier. (**b**) XPS Ag 3d spectra of samples [93]. (**c**) EXAFS fitting curve in R space of Ag_SA_-CNc [93]. (**d**) Fourier transform EXAFS spectrum [93]. (**e**) Wavelet transform EXAFS [93]. Copyright 2022, Wiley-VCH. (**f**) The difference of E_form_ between MoS_2_-NT and MoS_2_-slab [102]. (**g**) The relative work function for used models in (**h**) [102]. (**h**) The TDOS diagram of Mo-sub, Mo-sub-VS, Mo-sub+H, and Mo-sub-VS+H [102]. Copyright 2022, Wiley-VCH.

Scanning Transmission Electron Microscopy (STEM). STEM is an electron microscope that utilizes a field emission electron gun for imaging. By incorporating an aberration corrector, the spatial resolution of a STEM can achieve sub-micrometer levels, allowing for the detailed characterization and analysis of materials’ microstructure and fine chemical components at the nanometer and atomic scale. The electron beam generated by the field emission electron gun is focused into an atomic-scale electron beam spot through a sophisticated light collection system, serving as a highly precise electron probe for scanning the sample point by point under the guidance of the scanning coil. In contrast to parallel electron beam TEM, where an aggregated electron beam is scanned across the sample, STEM imaging involves a focused electron probe scanning the sample point by point. The detector in STEM receives either transmitted or elastically scattered electrons, which are then amplified and displayed on a fluorescent screen to produce bright field and dark field images. Various imaging modes, such as annular bright field (ABF), annular dark field (ADF), and high angle annular dark field (HAADF), capture scattered signals from different angles to reveal diverse material characteristics at the same location. HAADF imaging, in particular, excels due to the ability to detect electron signals scattered at high angles through incoherent scattering. The intensity of these scattered signals is directly related to the number of atoms and the square of the atomic number. Analysis of the intensity distribution in the image enables the identification of mono-heteroatoms in various crystal orientations. Notably, imaging 2D layerless materials with minimal overlap projections results in less distortion and improved STEM imaging performance [89]. In a study conducted by Yang et al., error-corrected high-angle dark field scanning transmission electron microscopy is used to examine Ag_1_/CN. As shown in Figure 7a [90], the resulting AC-HAADF-STEM plot displayed numerous small dispersed black dots, indicating the dispersed structure of Ag_1_/CN, in particular, AC here refers to spherical aberration correction [90]. 

#### 2.3.2. Spectroscopic Characterization Techniques

*X-ray Photoelectron Spectroscopy (XPS).* XPS is a surface analysis technique that provides compositional and structural information within the shallow region, specifically defined as a depth of less than 2 nm beneath the surface of a sample. XPS operates on the principle of using X-ray radiation to excite the innermost layers or valence electrons of atoms or molecules, causing them to emit photoelectrons. By irradiating the material’s surface with X-ray photons within an energy range of 1000–1500 eV, quantitative, and structural characterization of the material is achieved by analyzing the energy distribution of emitted photoelectrons and ohmic electrons. An electron spectrometer is utilized to collect data on the energy, angle, and intensity of these excited electrons [91]. XPS is also referred to as electron spectroscopy for chemical analysis (ESCA) due to its primary application in the chemical analysis of XPS. The resulting XPS spectra display the kinetic/binding energy of photoelectrons on the horizontal axis and the relative intensity (counts per second) on the vertical axis. This spectral information allows for the identification of elemental species based on characteristic peaks and the determination of peak shifts that signify changes in valence states. Moreover, XPS facilitates the assessment of heteroatom dispersion modes and the quantitative analysis of different elements or valence states, particularly in surface-loaded catalyst forms, offering enhanced accuracy in the quantitative analysis [92]. For instance, Huang et al. demonstrate the intermediate oxidation state of Ag SAs by examining the main valence states of Ag at 3d_5/2_ and 3d_3/2_ through XPS analysis (Figure 7b) [93].

*X-ray Adsorption Spectroscopy (XAS).* XAS is a powerful technique used to analyze the elemental composition, electronic states, and microstructure of materials by examining the changes in signals before and after exposure to synchrotron radiation X-rays [94]. The X-ray absorption coefficient (μ) of a material typically decreases with increasing X-ray energy, but there is a notable increase at the X-ray absorption edge, where the X-ray photon energy matches the ionization energy of the inner electrons in the atom [95]. X-ray absorption near-edge structures (XANES) exhibit strong oscillations approximately 10 eV before and 50 eV after the absorption edge, while extended X-ray absorption fine structure (EXAFS) shows continuous, weak oscillations extending up to approximately 1000 eV [96,97]. XANES can be further categorized into leading edge, edge, and trailing edge, each providing valuable information on the valence, coordination species of atoms, and local electronic structure of vacancy positions [97]. When processed using Fourier transform (FT) or wavelet transform (WT), the coordination number, bond length, and coordination structure of metal and non-metallic heteroatoms can be determined [98]. As seen in Figure 7c [93], Huang and colleagues conducted a study to analyze the local coordination environment and electronic structure of individual Ag atoms using XANES and EXAFS. The k_3_-weighted k-space exhibited two main peaks at approximately 1.56 Å and 2.41 Å, corresponding to the first and second shells coordinated with AgN and AgC, respectively (Figure 7c) [93]. In the case of Ag_SA_-CN, no peaks were detected for Ag-Ag coordination (2.64 Å) or Ag-O coordination (1.62 Å) in the R space, supporting the concept of atomically dispersed Ag species coordination with C and N in CN. Additionally, a wavelet transform EXAFS analysis was conducted to visualize structural details (Figure 7d) [93]. The EXAFS spectrum showed a single peak at 3.6 Å for AgN/C coordination in Ag_SA_CN, contrasting with signals from Ag foil and Ag_2_O (see Figure 7e) [93]. Analysis of the EXAFS spectrum revealed coordination numbers of 2.2 for AgN and 1.7 for AgC [93].

*Electron Paramagnetic Resonance (EPR).* EPR spectroscopy is a technique used to detect paramagnetic signals produced by the magnetic moments of unpaired electrons within vacancies. An electron, an elementary particle with mass and a negative charge can exhibit two types of motion: orbital motion around an atom’s nucleus and spin motion around its own axis. Unpaired electrons in atoms or molecules possess spin, defined as rotation around their own axis. The spins of these unpaired electrons interact with an external magnetic field, resulting in a specific energy level structure observed in EPR spectroscopy. Microwave radiation is employed to induce the unpaired electrons to transition between energy levels, producing a resonance signal. EPR utilizes the magnetic moments of unpaired electrons, particularly in free radicals where orbital moments have minimal impact, and the majority (over 99%) of the total magnetic moment is due to electron spin. Therefore, EPR is also referred to as “electron spin resonance” [99].

*Positron Annihilation Technique (PAT).* PAT is a non-destructive research method that utilizes positron annihilation radiation in condensed matter to provide insights into the internal microstructure, electron momentum distribution, and defect state of materials. The positron lifetime is the duration between the creation and annihilation, encompassing the entire lifespan within the material. The initial detection of positron generation is indicated by 1.28 MeV γ photons emitted by 22Na, while the annihilation event is marked by 0.511 MeV γ photons resulting from the interaction between positrons and electrons. By measuring the time delay between these two signals, the positron lifetime can be determined. Through monitoring the positron annihilation process, a lifetime spectrum is generated with a time resolution of around 200 ps at full-width half-maximum. Subsequently, lifetime parameters are calculated and fitted. The shortest lifetime (τ1) is linked to lattice defects, the intermediate lifetime (τ2) is attributed to vacancy aggregation, and the longest lifetime (τ3) is associated with annihilation events at surfaces or within large voids. By comparing the intensities of these lifetimes (I_1_, I_2_, and I_3_), researchers can discern the predominant types of vacancies and trends in vacancy distribution [100].

#### 2.3.3. Density Functional Theory (DFT)

DFT is a computational method used in quantum chemistry to analyze electron distribution by minimizing the system’s total energy. This approach clarifies the thermodynamic basis of catalytic properties and enables the prediction of materials’ electronic structures and energy band structures. DFT is also valuable for studying chemical reaction mechanisms, transition states, energy barriers, and related phenomena [101]. For example, Liu et al. employ two DFT computational models to examine the characteristics of flat Pt-doped 2H-MoS_2_ (MoS_2_ sheet) and nanotube configuration (MoS_2_ NT). The models consider scenarios where Pt atoms replace Mo sites (sub Mo), S sites (sub S), are positioned above Mo atoms (top Mo), or above vacant locations (top hollow). As shown in Figure 7f [102], the calculated energy (E_form_) for sub Mo formation in Pt/MoS_2_ sheets is found to be 0.33 eV, much lower than the vacancies in the MoS_2_ layer (2.33 eV), indicating that sub Mo formation via S vacancies (sub Mo VS) is more favorable in Pt/MoS_2_. Analysis of sub S formation revealed that Pt exhibits a stronger affinity for S defect sites within MoS_2_ [102]. The analysis of the adsorption and desorption dynamics of active catalyst sites in intermediate reactions can be used to investigate the relationship between the electronic configuration and the catalytic efficiency of catalysts. In the case of MoS_2_ NT, the presence of a lower energy state at the vacant peak compared to the Mo peak indicates enhanced stability for the HER. Comparisons between MoS_2_ NT and MoS_2_ layers show that the energy levels of sulfur and molybdenum in MoS_2_ NT are significantly lower than those in the MoS_2_ layer, suggesting superior catalytic activity for the HER. The energy levels of molybdenum (−0.055 eV) and the Mo peak (0.079 eV) in MoS_2_ NT are close to thermal equilibrium, allowing them to overcome absorption and emission barriers. After the absorption of hydrogen atoms at the sulfur cavity (referred to as Mo sub VS+H), the energy of Mo sub VS is lower than that of Mo sub. These findings are illustrated in Figure 7g [102].

The hydrogen adsorption energy on a metal surface is primarily determined by the interaction between a hydrogen intermediate orbital and a metal d-orbital. The degree of filling of these orbitals affects the key performance index of hydrogen adsorption energy (ΔG_H*_). Predicting the performance of transition metal (TM)-based catalysts in the HER can be achieved by calculating the projected density of states (PDOS) of the d-band, which indicates the position of the d-band center (Figure 7h) [102]. The DFT method, known for computational efficiency and scalability in handling complex systems, is commonly used. However, it may not provide entirely accurate results for certain systems like strongly correlated systems or high-temperature and high-pressure conditions. The accuracy of DFT calculations also depends on the choice of generalized functions and basis sets. Despite its limitations, the DFT method is still reliable for predicting qualitative trends [103].

## 3. Different Types of Two-Dimensional Supported Single-Atoms

### 3.1. Two-Dimensional Carbon-Based Supports

As the supporting materials of SACs, 2D carbon-based nanomaterials are increasingly used in heterogeneous catalysis due to their cost-effectiveness, excellent chemical stability, large specific surface area, and tunability at various scales [104], and are considered promising supports for atomically dispersed metal catalysts [105]. Liu et al. prepared an SAC named Co-CNG using a simple annealing process with g-C_3_N_4_ as the substrate and Co single-atom coupling. The as-prepared Co-CNG displays good HER performances in 1 M KOH, which attains only 47 mV to achieve the current density of 10 mA cm^−2^ with a Tafel slope of 44 mV dec^−1^. The Co-CNG has excellent stability with a four-time high mass activity as compared to the commercial Pt/C (Figure 8c) [106]. The excellent HER performances are attributed to the interaction between the edge sites of cobalt single atoms and Co-N. The Co-N model has a downshifted d-band center, a localized electronic structure around the Fermi energy, and a low free energy barrier, which can help improve the performance of HER [106]. Wei et al. discovered a novel electronic of new porous graphitic carbon nitride (g-CN) doped with dual transition metal atoms (Fe, Co, Ni, etc.), to conduct HER. These catalysts present benefits such as even hole distribution, high stability, and outstanding performance for electrocatalytic HER [107]. Co_1_/g-CN and Ni_1_/g-CN can drive HER over potential as low as 0.15 V and 0.12 V. Beyond commercial Pt and IrO_2_. The reason for the excellent performance of the catalyst is that the d-band center of the TM atom can act as an effective descriptor of the strength of the interaction between intermediates and TM/g-CN. Yang et al. create a carbon structure with a single vacancy capable of trapping Pt-SA to generate a Pt-C_3_ configuration (Figure 8d) [108]. The findings demonstrate that the well-coordinated Pt-C_3_ structure shows enhanced electron capture capacity and a reduced Gibbs free energy difference (ΔG), facilitating the hydrogen adsorption reduction and desorption process. Consequently, this configuration displays remarkable HER activity [108]. Isolated Pt atoms are captured onto defective graphene supports to prepare Pt-C_3_ coordinated structures (Pt@DG) by the annealing process (Figure 8e) [108]. Pt@DG shows good overpotentials of 30 mV and 37 mV at a current density of 10 mA cm^−2^, respectively, in 0.5 M H_2_SO_4_ and 1 M KOH. Meanwhile, the Tafel slope value of the Pt@DG is 53 mV dec^−1^.

Two-dimensional carbon-based nanomaterials possess a unique structure and significant surface area, making them ideal for supporting SACs [109]. The surfaces of 2D carbon-based nanomaterials can be easily modified to attach different coordinating atoms and generate various surface defects [110]. Consequently, 2D carbon materials hold great promise as support for SACs to conduct HER [111].

### 3.2. Two-Dimensional Layered Double Hydroxides (LDHs) Supports

LDHs exhibit a unique 2D layered structure that enables a wide range of surface electronic structure modulations [112]. Techniques such as single-atom doping, local structure tuning, and the addition of defect structures effectively enhance the performance of SA sites [113]. LDHs have attracted considerable attention for their ability to facilitate hydrolysis dissociation during the HER process, thereby promoting HER catalysis in alkaline environments [114]. Regulating LDHs with monatomic precious metal atoms can enhance HER performance, as the precious metal can act as the active site or regulate the electronic structure of the transition metal site in LDHs by surrounding the monatomic precious metal atoms [115]. LDHs are an excellent support for stabilizing SAs. Wang et al. develop a highly efficient catalyst by electrochemical phase transformation, denoted as Pt_SA_Co(OH)_2_@Ag nanowire, consisting of a 2D Co(OH)_2_ nanosheet grown on Ag nanowires anchored with Pt-SA. The as-prepared Pt_SA_Co(OH)_2_@Ag demonstrates a low overpotential of 29 mV in 1.0 M KOH to attain a current density of 10 mA cm^−2^ with the Tafel slope of 35.72 mV dec^−1^ and a mass activity 22.5 times higher than that of commercial Pt/C catalyst. The reasons for displaying such strong HER activity are that the Ag nanowire network provides a continuous electron transfer path through the metal active sites, contributing to extremely low charge transfer resistance (Rct, 0.7 Ω), and the graded nanostructure has a large electrochemical surface area for high atomic utilization efficiency and abundant mass transfer pathways for hydrogen generation and release. Moreover, the Co(OH)_2_-modified Pt site induces a local tip-enhanced electric field region around the Pt site and makes a greater contribution to H adsorption and H_2_O adsorption [116]. By modulating the electronic structure of 2D LDH surfaces, Hou et al. prepared a Ru_1_/D-NiFe LDH by electrochemical deposition, where a Ru-SA has been stabilized on defective nickel–iron layered double hydroxide nanosheets. Ru_1_/D-NiFe LDH provides an ultra-low hydrogen evolution overpotential of 18 mV at 10 mA cm^−2^ in 1 M KOH electrolyte, surpassing commercial Pt/C catalysts. The Tafel slope of Ru_1_/D-NiFe LDH is as low as 29 mV dec^−1^. The exceptional performances of Ru_1_/D-NiFe LDH are ascribed to the precise control over the local coordination environment of the catalytic active center and the presence of defects, which optimize the adsorption energy of reaction intermediates and promote the O-O coupling reaction on the Ru-O active site [117]. The 2D topographical properties of LDHs lead to specific characteristics at the edge positions when utilized as SA anchor localization points. Jin et al. achieve the precise construction of Pt single atoms on 2D layered Ni(OH)_2_ edges, named Pt-Ni(OH)_2_-E by in situ electrodeposition technology (Figure 9a) [118]. Compared with the monatomic Pt catalyst (Pt-Ni(OH)_2_-BP) anchored on the Ni(OH)_2_ base surface, PT-Ni(OH)_2_-E has higher electron affinity and higher intrinsic catalytic activity, which is conducive to the strong adsorption and rapid dissociation of water molecules. Therefore, the Pt-Ni(OH)_2_-E catalyst requires low overpotentials of 21 and 34 mV to achieve the current density of 10 mA cm^−2^ under both alkaline and neutral conditions with excellent stability (Figure 9b–d) [118]. The SACs can maintain the free atomic electronic structure, while the combination of LDHs and single-atom alloys (SAAs) can enhance the HER performances of SACs [119]. The NiIr SAAs onto NiFe-LDH named NiIr_SAA_-NiFe-LDH is prepared by Hu et al., which shows very excellent HER performance (Figure 9e). In 1.0 M KOH, the overpotential of NiIr_SAA_-NiFe-LDH is 28.5 mV at 10 mA cm^−2^, with a Tafel slope of 32.9 mV dec^−1^ (Figure 9f,g). The excellent performances of NiIr_SAA_-NiFe-LDH lie in the abundant hydroxyl groups on the surface of LDHs, which makes it easier for SAs to be loaded on the catalyst surface. Trivalent metal (M^3+^) ions are orderly dispersed by divalent metal (M^2+^) ions, and SAs can be uniformly dispersed on the catalyst surface, which enhances the performance of HER [120].

Further enhancements in performance can be achieved through regulatory and doping techniques while maintaining high HER activity through structural modifications [121]. Common techniques include constructing heterostructures on 2D LDH surfaces and elemental doping to improve metal–support interactions between LDHs and SA [122,123,124]. The improved performance of 2D LDHs support in facilitating the hydrolysis dissociation of SA catalysts has been well-documented [125]. In conclusion, LDHs demonstrate satisfactory performance in alkaline environments with SA modification and regulation [126]. However, to fully exploit the potential of LDHs across the entire pH spectrum, additional research efforts and investigations are needed to propel the field forward.

### 3.3. Two-Dimensional Transition-Metal Compounds Supports

Transition metal compounds have emerged as promising catalysts for water electrolysis in recent years [127]. Some compositions of these compounds have demonstrated HER activities comparable to platinum-group-metal (PGM)-based catalysts [128]. The 2D layered structures of transition metal compounds offer electrocatalysts with larger specific surface areas and higher densities of catalytic sites [34]. These structures exhibit lower in-plane electrical resistivity compared to that perpendicular to the basal plane, facilitating more efficient electron transport along the basal plane towards active edge sites [38]. The diverse electronic structures observed in transition metal compounds stem from variations in the d-orbital configurations of different transition metals. Furthermore, the introduction of SACs represents a significant advancement in multiphase catalysis, known for its remarkable catalytic efficiency, maximal atomic utilization, and well-defined structural characteristics [129]. Incorporating single atoms into the frameworks of transition metal compounds has propelled substantial progress in this field. Transition metal dichalcogenides (TMDs) have been extensively studied as potential single-atom supports. MoS_2_ as an SAC support has been widely used in the HER field. As a template of SACs, MoS_2_ has been widely used in the field of HER. Shi et al. report a high-purity MoS_2_ nanosheet and load Pt single atoms onto the nanosheets, named s-Pt/1T′-MoS_2_. The prepared s-Pt/1T′-MoS_2_ displays an excellent HER performance in 0.5 M H_2_SO_4_ which attains only 50 mV to achieve the current density of 10 mA cm^−2^ with the Tafel slope of 118 mV dec^−1^. This high-purity MoS_2_ nanosheet helps to disperse Pt single atoms on the catalyst surface, effectively suppressing atomic aggregation during HER [130]. Even after being heated to 800 °C, the catalyst remained highly stable and predominantly metallic (64% Cu). The Cu_0.39_MoS_2_ exhibits outstanding HER performance, with a low overpotential of 250 mV and a small Tafel slope of 52 mV dec^−1^ at 10 mA cm^−2^, which is attributed to changes in the electronic properties of MoS_2_ and enhanced conductivity (Figure 10a,b) [72]. He et al. prepared a novel deformed TMD monolayer known as curved MoS₂ (cMoS₂), finding that external compression can significantly reduce the metal adsorption energy (Figure 10c) [131]. The Gibbs free energies of Fe@cMoS₂ and Pt@cMoS₂ compressed to 16% and 4% are −0.02 eV and 0.03 eV, respectively [131]. Transition metal carbides (TMCs) are highly advantageous as ideal support materials for catalytic applications due to their exceptional corrosion resistance and efficient electron transfer capabilities, which contribute to sustained high activity and stability in intricate catalytic systems. Fu et al. combine monatomic nonmetallic phosphorus atoms to a monocrystalline Mo_2_C hexagonal nanosheet array supported by carbon sheets with exposed (001) faces by a pressure-assisted process to form an SAP-Mo_2_C-CS structure (Figure 10d) [132]. SAP-Mo_2_C-CS achieves an extremely low overpotential of 36 mV in 0.5 M H_2_SO_4_ with a Tafel slope of 38.1 mV dec^−1^ to obtain the current density of 10 mV cm^−2^ (Figure 10e,f) [132]. This kind of structure not only maintains a durable framework but also significantly boosts the electrocatalytic activity of the HER. The presence of a P-SA serves a dual purpose by generating localized electronic states through bonding with neighboring Mo atoms, DFT calculations have confirmed the stability of the P atom configuration [132]. Transition metal compounds typically contain highly electronegative anions, influencing the electron distribution of coordinated metal cations [133]. While the 2D surfaces of these compounds often exhibit limited catalytic activity, the exposed edge atoms demonstrate strong catalytic potential [134]. Surface modifications, such as vacancies and atomic doping, can significantly impact the electronic structure of these surfaces [135]. Therefore, when used as support for SAs, the interactions between the support surface and the SA can be precisely controlled.

### 3.4. Two-Dimensional MXenes and MBenes Supports

MXenes are 2D materials composed of carbide and nitride layers. Due to the superior activity and stability, excellent hydrophilicity, and multiple end groups (O, OH, F, etc.), MXenes have become a leading candidate for HER electrocatalysts. The material’s unique features such as strong structural integrity, chemical stability, excellent electrical conductivity, and large active surface area [96] have attracted great attention. Additionally, MXenes act as an effective support of SAs, offering diverse coordination environments and electronic states for metal atoms [136]. This interaction between the metals and the supports allows for the modification of both MXenes and the metal’s structural properties through the metal–support interaction (MSI) effect, enhancing MXenes’ chemical reactivity [137]. The development of the MSI effect provides MXenes with a versatile platform for catalyst design, facilitating the creation of a wide range of compositions and structural configurations. Liu et al. successfully utilize a linkage-assisted strategy to precisely deposit isolate monatomic ruthenium sites (Ru-SA) on nitrogen-doped Ti_3_C_2_T_x_ MXenes (N-Ti_3_C_2_T_x_) and named Ru_SA_-N-Ti_3_C_2_T_x_. The as-prepared Ru_SA_-N-Ti_3_C_2_T_x_ achieves remarkably low overpotentials of 27 mV and the Tafel slope is 29 mV dec^−1^ in 1 M KOH, 81 mV in 1 M PBS, and 23 mV with a Tafel slope of 42 mV dec^−1^ in 0.5 M H_2_SO_4_ at a current density of 10 mA cm^−2^. DFT calculations highlight a strong covalent interaction between the nitrogen site on Ru-SA and the Ti_3_C_2_T_x_ MXene supports as the key factor contributing to the superior catalytic performance and stability [138]. Zou et al. anchor Ru-SAs to stripped Ti_3_C_2_T_x_ by wet chemical impregnation (Figure 11a) [139]. The resulting Ru_SA_@Ti_3_C_2_T_x_ displays exceptional HER activity, particularly at high current density. It is worth noting that Ru_SA_@Ti_3_C_2_T_x_ can obtain high current densities of 1 and 1.5 A cm^−2^ at low overpotentials of 425.7 and 464.6 mV (Figure 11b,c) [139], respectively, and maintain strong stability, which indicates that it has practical application potential (Figure 11d) [139]. The presence of hydroxyl groups on the Ti_3_C_2_T_x_ surface is confirmed through the A_1g_ vibrational frequency shift in the Raman spectrum, providing important insights for mechanistic studies. The enhanced HER electrocatalytic performance of Ru_SA_@Ti_3_C_2_T_x_ can be attributed to the role of Ru-SAs in improving the adsorption and deionization of H_2_O, facilitating the efficient production of H_2_ [139]. The catalytic performance of MXenes can be enhanced by incorporating it with other active materials, thereby capitalizing on the exceptional support for SAs of MXenes. Ramalingam et al. employ the annealing method to support nitrogen and sulfur coordination Ru-SA on titanium carbide (Ti_3_C_2_T_x_) MXene to prepare Ru_SA_-N-S-Ti_3_C_2_T_x_. The as-prepared Ru_SA_-N-S-Ti_3_C_2_T_x_ shows excellent activity. The results show that the Ru_SA_-N-S-Ti_3_C_2_T_x_ catalyst attains an overpotential of 76 mV to achieve a current density of 10 mA cm^−2^ with a Tafel slope of 90 mV dec^−1^. DFT calculations suggest that Ru_SA_, in cooperation with the N and S sites on Ti_3_C_2_T_x_ MXenes, enhances the HER activity [140]. Given the affinity of MXenes for water, unique surface chemistry, and 2D structure, MXenes are well-suited for integration with materials such as SACs of transition or noble metals [141]. Platinum is renowned for its efficacy in catalyzing the HER and can be advantageously positioned as nanoparticles or individual atoms on a substrate. MXenes, with their favorable conductivity, exemplary hydration properties, and extensive surface area, could serve as an optimal substrate for platinum in this context [142]. This configuration precludes the agglomeration of atomically dispersed platinum nanoparticles, thereby facilitating the HER catalytic process. Zhang’s team utilizes a rapid thermal shock process to attach Pt-SAs to a Ti_3_C_2_T_x_ sheet, resulting in a Ti_3_C_2_T_x_-Pt_SA_ catalyst that exhibits impressive efficiency in the HER (Figure 11e) [84]. The Ti_3_C_2_T_x_-Pt_SA_ catalyst shows excellent hydrogen evolution performance in 0.5 M H_2_SO_4_, with attains only 38 mV to achieve the current density of 10 mA cm^−2^, the Tafel slope is 45 mV dec^−1^ (Figure 11f,g) [84]. DFT calculations suggest that this combination enhances the kinetics of hydrogen adsorption and desorption, leading to an improved HER process [84]. Electrocatalysts for the HER, typically based on precious metals like Pt or Ru, are highly efficient but costly, hindering large-scale production. To address this issue, researchers are exploring cost-effective alternatives, including non-precious metals like Ni, V, Mo, Fe, Cr, and W. Yun et al. develop a hybrid material by pyrolyzing Ni-SAC on a Ti_3_C_2_T_x_ MXene sheet and Fe-MOF. This approach resulted in a highly effective catalyst combining MXene, MOF, and SACs for hydrogen production. The incorporation of Ni-SAC onto MXenes through Ni-C coordination significantly improved the performance of the MXene-Fe-MOF hybrid catalyst. The catalyst shows a low overpotential of 52 mV in 0.5 M H_2_SO_4_ to attain the current density of 10 mA cm^−2^ (vs. RHE) with a stable Tafel slope of ~30 mV dec^−1^. The catalyst shows excellent superior stability when tested for long-term stability at a constant current density of 10 mA cm^−2^ by the concurrent method, with no attenuation of the current density for 27 h [143].

With the advent of graphene, more and more attention has been paid to single-layer 2D materials [144]. The researchers made transition metal borides (MBenes) by applying the idea of graphene to boron. MBenes are representative 2D layered materials known for their outstanding electrical conductivity, high melting point, remarkable physical hardness, low work function, and strong chemical resistance [145]. Yu et al. delve into the catalytic activity mechanism of hydrogen evolution in three distinct borene structures using the ab initio method, namely Pmmm, PmMN, and BM. Among these structures, the borene Pmmn configuration exhibits superior catalytic hydrogen evolution activity compared to the Pmmm and triangular BM structures. The computed electronic structure unveils that hydrogen in the PmMN structure of borene-induced band migration from the valence band to the conduction band, resulting in a metal-like behavior in the adsorption state that promotes catalytic hydrogen evolution. At θ = 2/32 hydrogen coverage, Pmmn borene exhibits excellent hydrogen evolution activity, and the Gibbs free energy is only −0.003 eV [146]. Metal borides have rich compositions and diverse structures, which makes metal borides have more possibilities in electrocatalytic hydrogen evolution [147]. Zhang et al. conducted a study on the HER catalytic performance of Mo_2_B_2_ catalysts with transition metal atoms incorporated in the Mo vacancy through first-principles calculations. The results show that these catalysts exhibit high metallic conductivity, leading to improved charge transfer during electrocatalysis. Ni@Mo_2_B_2_ has ΔG_H_ values of −0.09 and 0.12 eV at 1/4 and 1/9 hydrogen coverages, respectively, indicating that Ni@Mo_2_B_2_ still exhibits high HER catalytic activity under high hydrogen coverage. Experimental testing of Mo_2_B_2_ loaded with single-atom transition metals demonstrated that Ni@Mo_2_B_2_, in particular, displayed effective performance for HER [148]. Furthermore, the low electronegative of boron (B) in MBenes can lead to a singular electronic state of Pt-B interaction, thus conferring a near-zero valence state of Pt SA to provide a catalytic behavior comparable to that of metallic Pt surfaces upon the adsorption of H* and subsequent generation of H_2_ during HER [149]. Park et al. report a layered ternary transition metal boride nanosheet, MoAl_1-x_B, prepared by the selective etch extension method, which has a rich exposition of basal planes and Mo vacancy defect sites and can fix Pt single atoms (SA) by adsorption and doping behavior. The as-prepared Pt-MoAl_1−x_B exhibits Pt-like HER kinetics with enhanced overpotential values of 32 and 18 mV at 10 mA cm^−1^ in alkaline and acid media, respectively. The results show that MoAl_1−x_B can form a strong interaction with Pt SAs, resulting in the charge density and d-electrons enrichment of Pt atoms, and has excellent catalytic activity and stability. Therefore, it has the potential to be used as a highly efficient HER catalyst for practical electrolysis [149]. Wang et al. fabricate an ultra-thin hexagonal Mo_3_B film with a thickness of 6.48 nm on the Mo foil over a large area by chemical vapor deposition. The first-principles calculations show that the ultrathin Mo_3_B film is metallic, which facilitates rapid electron transport along the active edge of the thin film, improving HER activity [150]. In fact, binary WB_2_, which also contains folded boron layers, is also less active than two-dimensional hexagonal MoB_2_, despite containing the more reactive transition metal W [147]. Therefore, Wang et al. propose a two-dimensional tungsten boride (WB_4_) lattice, in which the Gibbs adsorption free energy (ΔG_H_) of atomic hydrogen tends to an ideal value (0 eV) at 3% strain state and has HER catalytic performance comparable to that of metal borides. The WB_4_ lattice produces multiple Dirac cones around the Fermi level and transfers electrons in all directions throughout the structure at considerable Fermi speeds. What is more, the p-orbitals of borophene subunits in the WB_4_ lattice can modulate the d-band center together with the d-orbitals of W for good HER performance [151]. Boron and nitrogen are the two closest neighbors of carbon, and the outermost electrons of the two elements are 3 and 5, one less and one more than the outermost electrons of carbon, respectively. The combination of boron and nitrogen can also form a hexagonal boron nitride (h-BN) two-dimensional material with a honeycomb structure. Saha et al. designed a metal-free 2D structure of graphene and boron nitride (h-BN) by the annealing method. The two-dimensional stacking of two different layers yields rich interfaces as well as huge electrochemical active sites. As a result, the graphene/h-BN stacked structure has a very low hydrogen evolution overpotential of 28 mV to attain the current density of 10 mA cm^−2^ in 1 M KOH. The graphene/h-BN shows a very low Tafel slope of 25 mV dec^−1^, confirming the fast reaction kinetics [152].

The surface of MXenes contains heteroatoms like N and S, along with various surface functional groups [153], giving it a natural bonding advantage with metal SACs and strong anchoring capabilities [154]. MBenes have a unique two-dimensional hexagonal honeycomb structure, which gives it the band structure of a Dirac cone and novel quantum effects, it shows excellent catalytic activity against HER. As a promising 2D material, MXenes and MBenes hold great potential as supports for SACs.

### 3.5. Other 2D Materials Single-Atom Supports

Two-dimensional materials are extensively studied as potential electrocatalysts due to their layered structures and unique electronic properties. Various materials can be converted into 2D layered structures, serving as supports for SAs to explore electronic interactions in depth. Moreover, tailored morphological characteristics can be integrated into 2D materials to enhance catalytic performance [155]. Chang et al. prepare the B/TM/B (TM = Co, Ni, Cu, Pd) intercalation structure, a novel 2D configuration where transition metal atoms are embedded in a bilayer boride (Figure 12a) [156]. This structure demonstrates energy stability, structural stability, and thermal stability, withstanding temperatures of at least 1300 K. Variants like B/Cu_x_/B, B/Pd_x_/B, and B/Al_x_/B, with different metal coatings, exhibit very low HER free energies ranging from −0.162 to 0 eV. The electron energy levels in these systems are approximately 179 eV, −0.134 to 0.183 eV, and −0.082 to 0.086 eV, respectively, comparable to Pt. The B/TM/B system with a 2D intercalation structure displays good electrical conductivity, high structural and thermal stability, low intercalation resistance, a straightforward water decomposition process, excellent defect-dependent catalytic properties, cost-effectiveness in raw materials, and non-corrosive attributes.

Various surface defects have been identified, with dense defects at crystal dislocations of heterogeneous structure interfaces garnering significant attention from researchers. Pt-SA supported on 2D NiO/Ni heterojunction nanosheets presents notable advantages [157]. The presence of numerous vacancies induced by crystal dislocations and phase transitions at the NiO/Ni interface creates effective sites for capturing Pt-SA. Experimental findings and theoretical calculations confirm that Pt atoms predominantly coordinate with one oxygen atom and five nickel atoms, anchoring at the Ni/NiO interface. The coupling of Pt single-atom and NiO/Ni heterostructure makes the binding ability of hydroxide ion (OH*) and hydrogen ion (H*) adjustable, which effectively adjusts the dissociation energy of water, promotes the transformation of H*, and accelerates the alkaline hydrogen evolution reaction (Figure 12b,c) [157]. Therefore, the fabricated Pt_SA_-NiO/Ni catalyst shows high alkaline hydrogen evolution properties and the mass activity of Pt is quite high at 100 mV overpotential, with 20.6 A mg^−1^, which is significantly better than the reported catalyst (Figure 12d,e) [157]. Researchers are exploring a wide range of 2D support materials, with some emerging supports being used as SA supports, leading to unexpected outcomes. Carbon-based covalent organic framework (COF) materials have particularly piqued interest because of their distinctive pore structures and plentiful surface functional groups. By employing electrochemical methods to load single-atom Pt onto a 2D nitrogen-rich graphene-like COF (NGA-COF@Pt) [158], researchers successfully prepared a Pt-based SAC at room temperature, eliminating the need for conductive agents or pyrolysis. Utilizing a bottom-up approach, Pt single atoms are uniformly anchored on the highly π-π conjugated graphene-like COF with a Pt loading of approximately 2.66 wt.%. The study shows that using a COF as a current collector improved charge transfer and enhanced metal–support interactions for Pt-SA catalysts. NGA-COF@Pt shows overpotentials of 13, 17, and 366 mV, respectively, to achieve the current density of 10 mA cm^−2^ in acidic (0.5 M H_2_SO_4_), alkaline (1 M KOH) and neutral conditions, with a Tafel slope of 21.88 mV dec^−1^ (Figure 12f) [158]. The HER performance of the experimental sample is better than that of the commercial benchmark Pt/C. This surpasses the performance of most catalysts reported in the literature.

The use of 2D support materials for SACs has advanced our understanding of catalyst-support interactions and electronic dynamics. This knowledge is crucial for improving the effectiveness and durability of SACs. The growing variety of SACs necessitates more sophisticated synthesis techniques and exploration of different strategies for preparing 2D support materials and loading SAs. Table 1 provides representative SACs HER properties and preparation methods, aiming to highlight the application prospects of 2D SACs and stimulate the progress of current synthesis methods and the development of new strategies. 

## 4. Manipulating the Microenvironment of SAs on Metal–2D Nanomaterials

Understanding the interaction between the metal and the support in SACs is a critical area of research. The support plays a key role in dispersing and immobilizing the active metal components, thereby influencing the catalyst’s performance [159]. In particular, the interaction between active sites and 2D supports can have a significant impact. Recent advancements in characterization techniques and theoretical computational methods have enhanced researchers’ understanding of these interactions and their effects on catalytic performance and mechanisms. In situ techniques now allow for real-time monitoring of reaction pathways, advancing studies on the catalytic activity, stability, and mechanisms of SACs. The concept of metal–support interactions has evolved to encompass changes in interfacial structure [160], coordination [161], charge transfer [39], anchoring effects [142], and more. Interactions between metal and support in electrocatalytic technology are categorized into strong metal–support interactions (SMSI), oxide metal–support interactions (OMSI), covalent metal–support interactions (CMSI), and electronic metal–support interactions (EMSI) [162]. The focus on regulating surface and interfacial chemical properties, particularly the electronic structure, has become crucial in electrocatalyst design, especially for hydrolysis reactions (HER). The activity and stability of electrocatalysts are closely linked to characteristics like chemically active sites, adsorption capacity, and electronic density of states on the catalyst surface. By precisely adjusting these properties, the performance and durability of electrocatalysts can be significantly enhanced, representing a key aspect of catalyst design and optimization [163]. This article provides a detailed overview of interactions between 2D supports and SA active sites, exploring their principles for improving HER performance and stability. The insights offered aim to guide the design of SA catalysts supported on 2D materials.

### 4.1. Dopant Coordination Effect

Introducing non-metal atoms such as nitrogen or sulfur into 2D support materials as coordination atoms can significantly regulate the metal–support interactions (MSIs) of SACs [164]. Nitrogen doping in carbon materials, for example, can increase the positive charge on adjacent carbon atoms due to nitrogen’s high electronegativity [165]. Pyridinic and pyrrolic nitrogen can offer more active metal anchoring sites through p-d orbital hybridization (Figure 13a). The coordinated N atoms in Fe-N_3_C_2_-C and Fe-N_4_-C have the same number of charges, and the symmetric electronic state distribution can be well demonstrated [166]. As seen in Figure 13b [167], graphitic nitrogen can boost catalytic activity by reducing the band gap of the carbon matrix via an upward shift of the highest occupied molecular orbital (HOMO) [167,168]. Moreover, sulfur atoms, despite being challenging to coordinate alone due to their larger atomic radius and weak charge transfer, can induce higher asymmetric spin and charge density through co-doping/multi-doping effects, leading to increased defect sites and catalytic sites in the carbon framework, thereby enhancing the stability and catalytic performance of SACs (Figure 13c) [169]. Additionally, positively charged boron (B) atoms can serve as Lewis acid sites to accept electrons from the carbon support, inducing p-type conductivity in carbon and indirectly modulating the d-band structure and oxidation state of metal sites (Figure 13d,e) [170]. The incorporation of non-metal coordination atoms on the surface of metal compounds also has a positive impact on the electronic structure regulation of single atoms. Various atomic doping strategies on the surfaces of 2D carbon-based or non-carbon-based materials can result in diverse effects (Figure 13f) [170].

**Figure 14:**
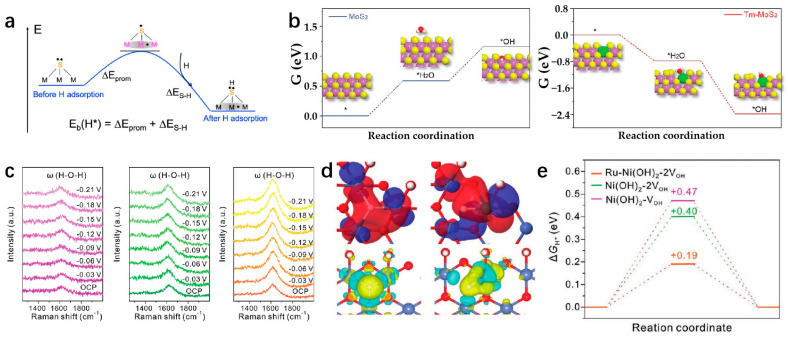
(**a**) Physical model for H-S bond formation [173]. Copyright 2020, Wiley-VCH. (**b**) Free energy diagram for MoS_2_, and Tm-MoS_2_ (* Represents the adsorption site) [174]. Copyright 2024, Wiley-VCH. (**c**) In situ Raman spectra at different potentials [177]. (**d**) Top views of the hollow site orbital configurations and charge density differences for H adsorption on Ni(OH)_2_-2V_OH_ (left) and Ru-Ni(OH)_2_-2V_OH_ (right) [177]. (**e**) Diagram of hydrogen adsorption free energy (∆G_H*_) [177]. Copyright 2023, Wiley-VCH.

**Figure 1:**
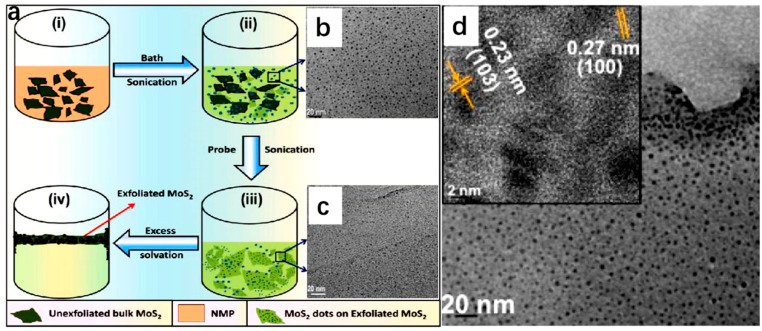
(**a**) Schematic diagram of a synthesis procedure for MoS_2_ quantum dots interspersed in MoS_2_ nanosheets using a liquid stripping method in 1-methyl-2-pyrrolidone solution [61]. (**b**) TEM images of MoS_2_ quantum dots of size ~2 nm formed through the bath sonication process [61]. (**c**) TEM image of MoS_2_ quantum dots scattered in a stripped MoS_2_ nanosheet [61]. (**d**) Transmission electron microscope image of MoS_2_ quantum dots on MoS_2_ nanosheets. The above HRTEM image of a MoS_2_ quantum dot shows the respective lattice spacing [61]. Copyright 2014, American Chemical Society.

**Figure 3:**
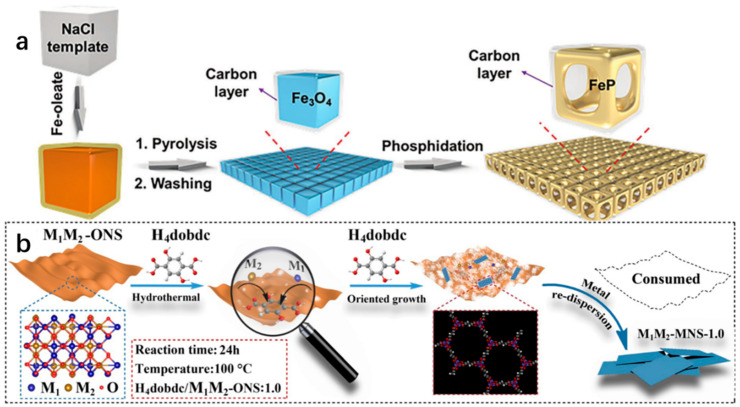
(**a**) Schematic showing the synthesis of 2D FeP nanoframe superlattices [73]. Copyright 2022, Wiley-VCH. (**b**) Schematic diagram of the 2D oxide sacrifice approach transformation process of M-MnS formed by metal oxide nanosheets and H_4_dobdc ligands, H_4_dobdc refers to 2, 5-dihydroxyterephthalac acid [74]. Copyright 2019, Wiley-VCH.

**Figure 4:**
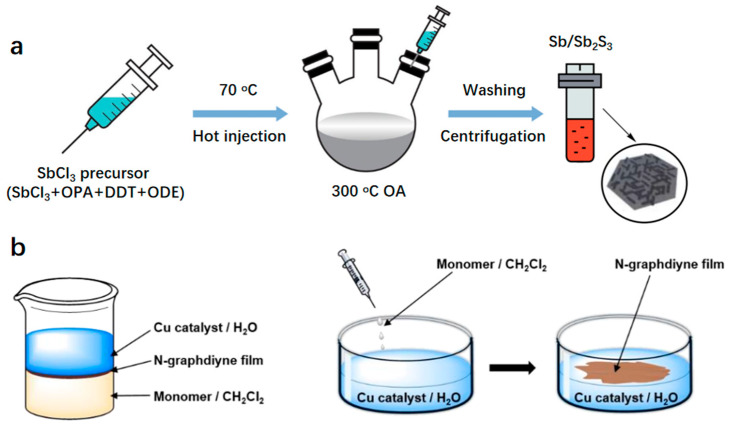
(**a**) Sb/Sb_2_S_3_ sample preparation process sketch [75]. Copyright 2023, Springer Nature. (**b**) Diagram of sample preparation at the liquid/liquid and gas/liquid interfaces [76]. Copyright 2018, American Chemical Society.

**Figure 6:**
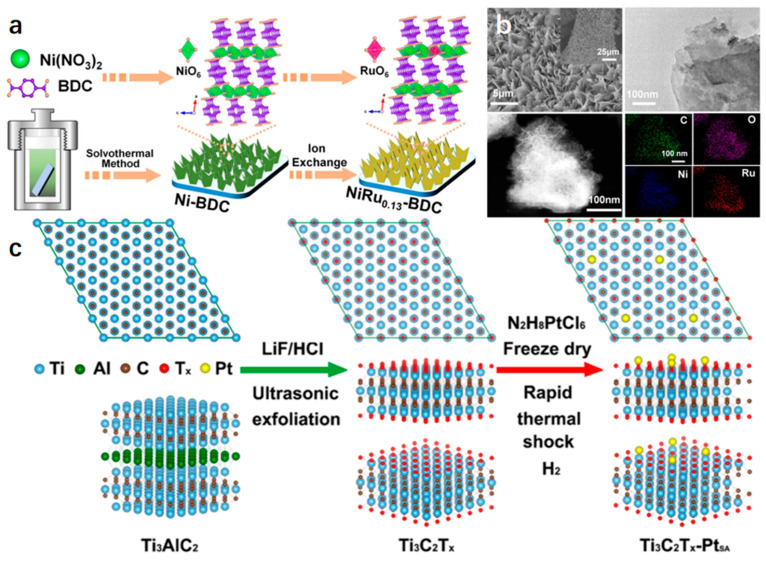
(**a**) Schematic illustration for the preparation of NiRu_0.13_-BDC catalyst [83]. (**b**) SEM images of NiRu_0.13_-BDC, HAADF-STEM images, and corresponding STEM-EDS mapping of NiRu_0.13_-BDC [83]. Copyright 2021, Springer Nature. (**c**) Schematic diagram of building Pt-SA on a Ti_3_C_2_T_x_ monolayer [84]. Copyright 2022, American Chemical Society.

**Figure 8:**
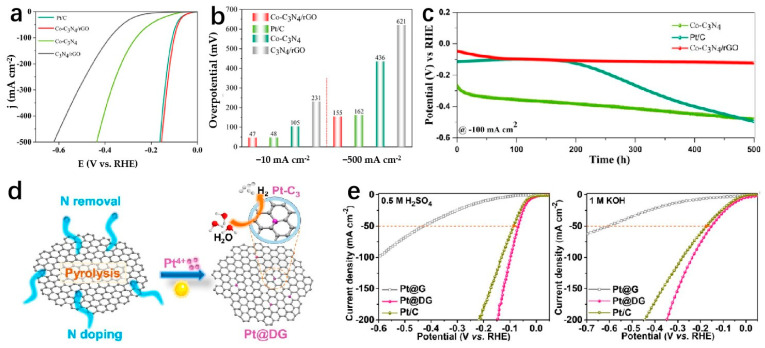
(**a**) LSV curves of Co-C_3_N_4_/rGO, Co-C_3_N_4_, C_3_N_4_/rGO, and Pt/C in 1.0 m KOH at a scan rate of 5 mV s^−1^ [106]. (**b**) Comparison of the overpotential required to reach a current density of 10 (500) mA cm^−2^ [106]. (**c**) Time-dependent overpotential curve of Co-C_3_N_4_/rGO, Co-C_3_N_4_, and Pt/C obtained at a constant cathodic current density of 100 mA cm^−2^ under 1.0 M KOH condition [106]. Copyright 2022, American Chemical Society. (**d**) A single vacancy trapping atom Pt is synthesized in a carbon matrix (defective graphene) to form the Pt-C_3_ configuration [107]. (**e**) Polarization curves of Pt@G, Pt@DG, and Pt/C with a scan rate of 10 mV s^−1^ [107]. Copyright 2022, American Chemical Society.

**Figure 9:**
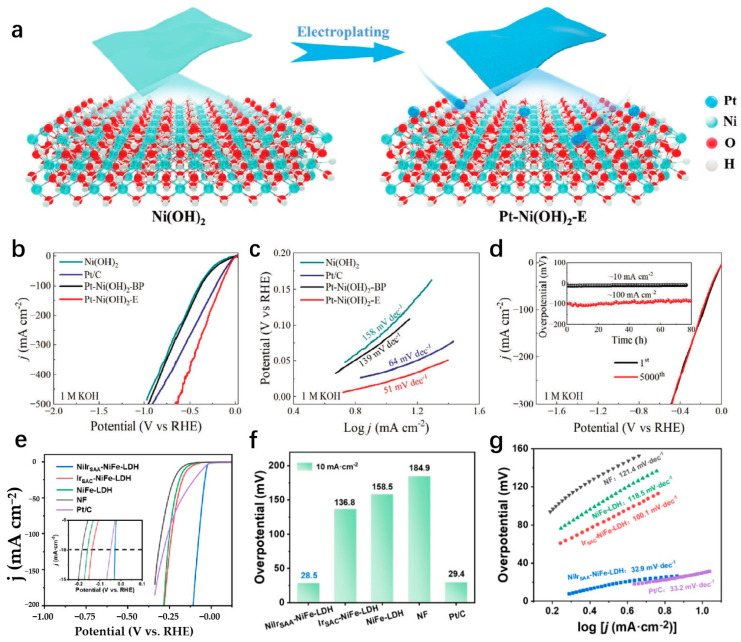
(**a**) Schematic illustration of the preparation for the Pt-Ni(OH)_2_-E [118]. (**b**) HER polarization curves of Ni(OH)_2_, Pt/C, Pt-Ni(OH)_2_-BP, and Pt-Ni(OH)_2_-E in 1.0 m KOH [118]. (**c**) Corresponding Tafel slope originated from LSV curves [118]. (**d**) Stability test of Pt-Ni(OH)_2_-E through potential cycling, before and after 5000 cycles in 1.0 m KOH. Inset: trigonometrical curves. HER performance of the as-prepared electrocatalysts and references in 1 M KOH [118]. Copyright 2023, Wiley-VCH. (**e**) LSV curves [120]. (**f**) Comparison of overpotential at 10 mA cm^−2^ [120]. (**g**) Tafel plots [120]. Copyright 2023, American Chemical Society.

**Figure 10:**
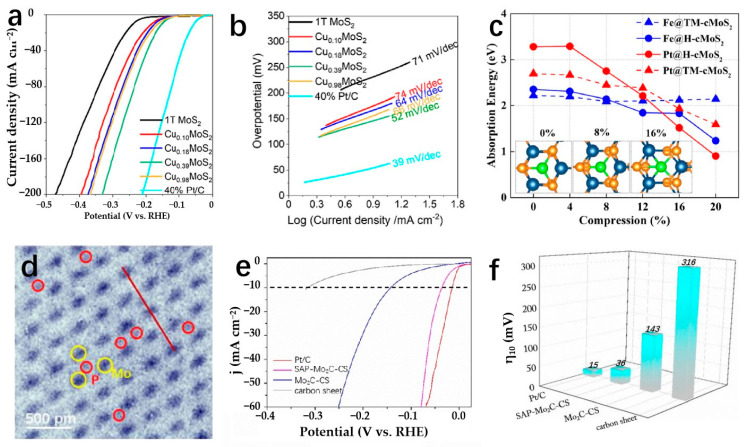
(**a**) HER LSV polarization curves of the as-prepared samples tested in 0.5 M H_2_SO_4_ solution [72]. (**b**) Corresponding Tafel slopes derived from HER LSV [72]. Copyright 2023, Wiley-VCH. (**c**) The adsorption energy of metal atoms on neutral cMoS_2_ as a function of compressions. The insets represent the top view of M@cMoS_2_ at different compressions [131]. Copyright 2021, Nanomaterials. (**d**) HAADF-STEM image of SAP-Mo_2_C-CS [132]. (**e**) HER polarization curves and corresponding η_10_ [132] (**f**), Tafel plots [132]. Copyright 2020, Wiley-VCH.

**Figure 11:**
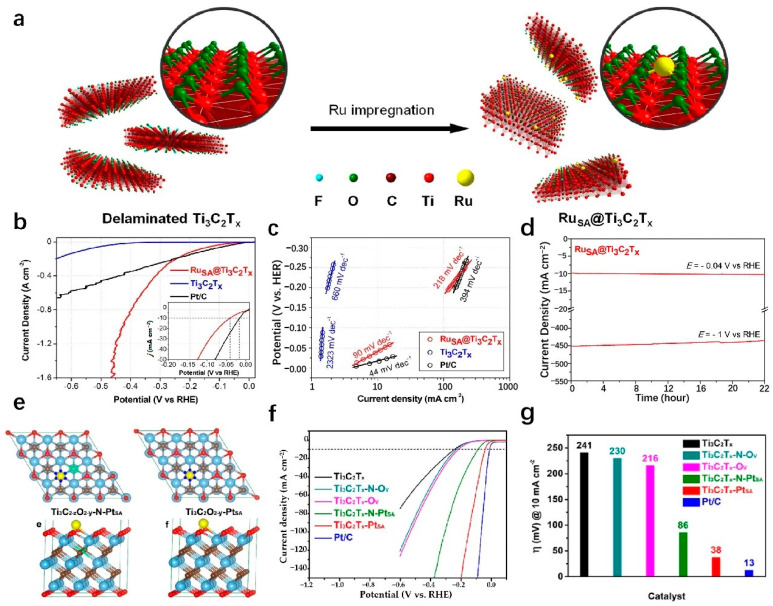
(**a**) Schematic illustrating fabrication procedure of Ru_SA_@Ti_3_C_2_T_x_ [139]. (**b**) iR_initial_-compensated linear sweep voltammetry (LSV) curves at a scan rate of 10 mV s^−1^ in 1.0 M KOH electrolyte [139]. (**c**) Corresponding Tafel plots under different potential ranges for Ru_SA_@Ti_3_C_2_T_x_, Ti_3_C_2_T_x_ and Pt/C [139]. (**d**) Chronopotentiometric stability of Ru_SA_@Ti_3_C_2_T_x_ under a constant potential of 40 mV and 1 V versus RHE [139]. Copyright 2022, Wiley-VCH. (**e**) Top and side views of the atomic structure of Ti_3_C_2_O_2_, Ti_3_C_2-z_O_2-y_-N-Pt_SA_, and Ti_3_C_2_O_2-y_-Pt_SA_. HER performances of different catalysts were measured in 0.5 M H_2_SO_4_ solution [84]. (**f**) LSV curves [84]. (**g**) Overpotentials at 10 mA cm^−2^ [84]. Copyright 2022, American Chemical Society.

**Figure 12:**
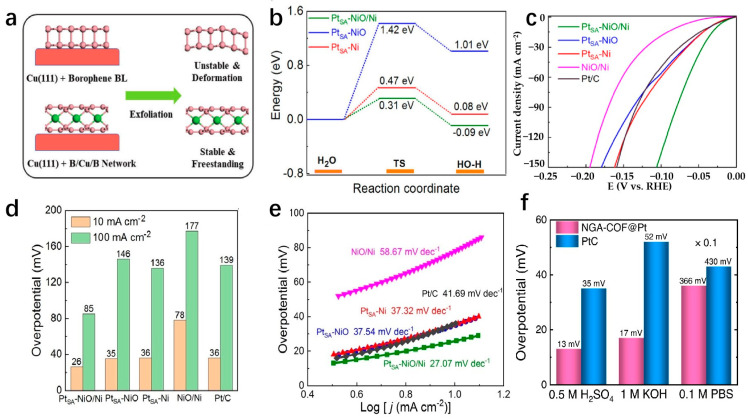
(**a**) Supported and freestanding diagrams of borophene bilayer and B/Cu/B [156]. Copyright 2023, Wiley-VCH. (**b**) Calculated energy barriers of water dissociation kinetic [157]. (**c**) HER polarization curves of Pt_SA_-NiO/Ni, Pt_SA_-NiO, Pt_SA_-Ni, NiO/Ni, and Pt/C in 1-M KOH electrolyte [157]. (**d**) The comparison of overpotentials required to achieve 10 and 100 mA cm^−2^ for various catalysts [157]. (**e**) Corresponding Tafel slope originated from LSV curves [157]. Copyright 2021, Springer Nature. (**f**) Overpotentials at 10 mA cm^−2^ for Pt/C and NGA-COF@Pt [158]. Copyright 2024, Springer Nature.

**Figure 13:**
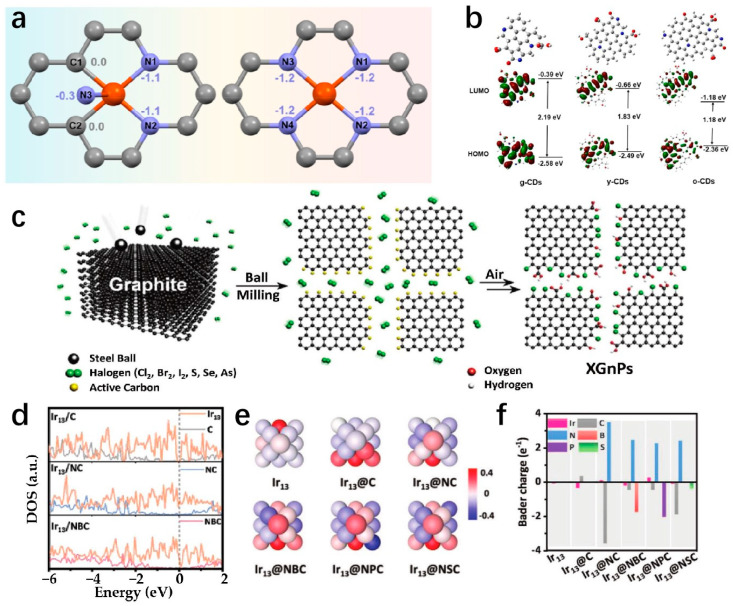
(**a**) Bader charge numbers of coordination atoms of Fe-N_3_C_2_-C and Fe-N_4_-C [166]. Copyright 2022, American Chemical Society. (**b**) The HOMO of graphite and nitrogen decreases the band gap of carbon matrix [167]. Copyright 2023, American Chemical Society. (**c**) A schematic representation for the ball milling approach to edge-doped graphitic platelets with different dopants [169]. Copyright 2018, Wiley-VCH. (**d**,**e**) Bader charge analysis projected to Ir_13_ cluster [170]. (**f**) DOS for Ir_13_, Ir_13_@C, Ir_13_@NC, Ir_13_@NBC [170]. Copyright 2022, Wiley-VCH.

To address the varied performance demands of catalysts, the introduction of multi-atomic co-doping can create synergistic effects. The functionalized MXenes surface, with abundant coordination atoms, fosters strong coordination interactions between these atoms and the single atoms. The study showcases the confinement of Ir-SA within porous, heteroatom (N, S) co-doped Ti_3_C_2_T_x_ [171]. XAS unveils the in situ bridge structure connecting Ir atoms and N, S atoms, promoting the effective dispersion of Ir atoms within the N, S doped porous Ti_3_C_2_T_x_. Furthermore, DFT calculations demonstrate that the N, S co-doped Ti_3_C_2_T_x_ support can extract electrons from Ir-SA, triggering charge rearrangement in the interface region of the Ti_3_C_2_T_x_ support, consequently enhancing the HER activity. Two-dimensional transition metal sulfides, such as MoS_2_, are recognized as promising electrocatalysts due to their unique structure and electronic properties, particularly in the context of the HER [20]. However, their catalytic activity on the basal plane (S-layer) is often limited due to poor electron transfer properties in these regions [172]. Single-atom doping is a viable strategy to activate these inactive sites by introducing foreign atoms to enhance HER performance (Figure 14a) [173]. For example, doping with metallic atoms like Fe, Co, Ni, and Ag can modify the electronic structure of MoS_2_, thereby increasing the HER activity. This form of doping can generate new active sites and potentially optimize proton adsorption and hydrogen atom formation by modifying the catalyst’s electronic properties. Incorporating single Tm atoms into MoS_2_ creates oxygen-affinitive Tm sites and forms asymmetric [Mo-S-Tm] unit sites, which enhance water adsorption and dissociation for alkaline HER. Characterization and theoretical calculations indicate that the strong electronic interaction between Tm-SA and Mo-S sites significantly influences the active S sites, resulting in a more favorable position for hydrogen adsorption-free energy (∆G_H_) (Figure 14b) [174].

Significant improvements in the HER performance of materials like MoS_2_ and other transition metal sulfides can be achieved through single-atom doping and defect engineering [175,176]. This approach can also be applied to other materials. For instance, in the case of the HER-inactive material Ni(OH)_2_, researchers used low-temperature plasma activation in combination with Ru SAs doping to activate HER activity and stability. As shown in Figure 14c [177], experimental tests and in situ Raman characterization showed that plasma activation and Ru SA doping could enhance water adsorption and dissociation on Ni(OH)_2_. Theoretical calculations suggested that Ru atoms near the β-Ni(OH)_2_/Ni-Ru SAs structure possess more delocalized electrons (Figure 14d) [177], which can have a significant impact on hydrogen adsorption reactions (Figure 14e) [177]. This indicates that Ru SA doping can modify the electronic structure of Ni(OH)_2_, thereby influencing the catalytic performance. Surface doping of 2D support can effectively change the electronic structure of the loaded surface. By doping with suitable heteroatoms, the interaction between the support and active sites is altered, leading to an enhancement in the catalyst’s HER performance [177].

### 4.2. Coordination Structure Effect

**Figure 15:**
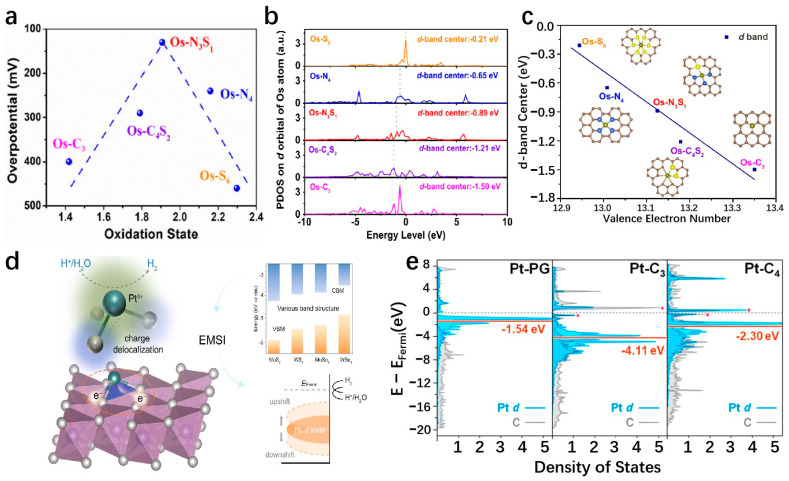
(**a**) ΔG_H*_ as a function of oxidation state of Os SACs [182]. (**b**) Projected density of state for d orbital of Os [182]. (**c**) The relationship between the d-band center and the number of valence electrons of Os-SA [182]. Copyright 2022, Springer Nature. (**d**) 2D TMDs modulate the electronic structure of Pt-SA by charge delocalization [183]. Copyright 2021, Springer Nature. (**e**) PDOS for d-band, C band (gray wire), and d band Pt (blue wire) [108]. Copyright 2022, American Chemical Society.

The local coordination structure of SAs is crucial for understanding the relationship between catalyst structure and performance [178,179,180]. The interaction between SAs and the support allows for adjusting the local electronic states of SAs, including their oxidation state and coordination number, which in turn affects catalytic performance [181]. The impact of oxidation state on activity is particularly important for enhancing catalytic performance. Researchers have identified a volcano relationship between the oxidation state of Os-SA catalysts and their activity in the HER. Initially, a range of Os-SA catalysts with oxidation states from +0.9 to +2.9 were synthesized by modifying coordination environments like Os-N_3_S_1_, Os-N_4_, Os-S_6_, Os-C_3_, and Os-C_4_S_2_ (Figure 15a) [182]. The relationship between oxidation state and HER activity follows a volcano pattern, peaking at an experimental oxidation state of +1.3 (Os-N_3_S_1_). Mechanistic studies suggest that as the oxidation state increases, hydrogen adsorption on Os strengthens due to higher energy levels and a decrease in the occupancy of the antibonding state of the Os-H bond until it becomes unoccupied (Figure 15b) [182]. Further increases in the oxidation state lead to weaker hydrogen adsorption due to reduced occupancy of the Os-H bonding state (Figure 15c) [182]. Therefore, the oxidation state plays a critical role in regulating activity in SACs. As depicted in Figure 15d [183], four Pt-SA catalysts were developed on various 2D TMDs supports (MoS_2_, WS_2_, MoSe_2_, and WSe_2_) utilizing site-specific electrodeposition technology as model systems. In acidic conditions for the HER, the catalytic activity significantly increases as the oxidation state of Pt-SA decreases due to a decrease in hydrogen binding energy. Conversely, in alkaline conditions, Pt-SA with an optimal oxidation state (approximately +2) demonstrates excellent catalytic activity by striking a balance between Pt-H interaction for hydrogen evolution and Pt-OH interaction for water dissociation. The tetracoordinated Pt-SA catalyst has shown promising performance in catalyzing HER, but recent studies have revealed that tricoordinated transition metal SACs may outperform tetracoordinated structures in certain reactions. A graphene-embedded Pt catalyst with Pt-C_3_ coordination has been proposed [183]. As shown in Figure 15e [108], DFT calculations suggest that the d-band center of the Pt-C_3_ coordination structure is lower than that of the pristine graphene-adsorbed Pt-SA structure and the Pt-C_4_ coordination structure, leading to weaker hydrogen adsorption and facilitating H_2_ evolution. Electrochemical experiments indicate that the Pt-C_3_ SAC achieves low overpotentials of only 0.03 and 0.037 V for HER under acidic and alkaline conditions, respectively, surpassing Pt/C. The optimal oxidation state and coordination may vary across different systems, but there exists a correlation between the local coordination environment of SAs and the catalytic performance of the catalyst.

### 4.3. Defect Engineering

**Figure 16:**
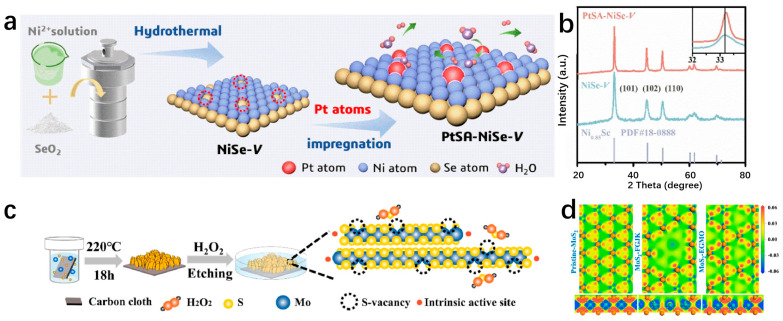
(**a**) Schematic illustration for the synthesis of Pt_SA_-NiSe-V [190]. (**b**) XRD patterns of samples [190]. Copyright 2023, Wiley-VCH. (**c**) Schematic of the chemical etching process to introduce single S-vacancies [193]. (**d**) Top view and side view electron density difference maps [193]. Copyright 2020, American Chemical Society.

Introducing defects into the support can directly alter the surrounding electronic structure [184,185,186]. The electronic perturbation of monatomic species by different local electronic structures can effectively modulate their d-band centers and, hence, their catalytic properties [187,188,189]. In a study involving cation vacancy-loaded SAs, a nickel selenide support enriched with cation vacancies was prepared using a hydrothermal method, and platinum single atoms were loaded using an impregnation method and named Pt_SA_-NiSe-V, as illustrated in Figure 16a,b [190]. The low electron density regions of the support anchored the high electron density platinum single atoms. At the cation vacancies, Pt formed Pt-Se bonds with the highly electronegative Se, accelerating electron transfer. Additionally, the good fixation provided by the cation vacancies ensured the long-term stability of the Pt-SA catalyst, with the electrode maintaining a high current density of 390 mA cm^−2^ and a retention rate of 99% over 80 h. Vacancies generate an additional attraction to the loading of monoatomic species, causing monoatomic species to specifically adsorb onto the vacancies. It is widely believed that vacancies have a strong effect on stabilizing monoatomic species. Vacancy defects are subtle imperfections that can actually improve the catalytic activity of catalysts. These defects, which are a special type of catalytic active center, have garnered significant attention due to their unique atomic arrangement and high distortion compared to regular lattices [191,192]. Through a gentle H_2_O_2_ chemical etching process, S-SA vacancies are evenly distributed on the surface of MoS_2_ nanosheets. These vacancies serve as efficient catalysts for the HER, allowing researchers to explore the correlation between structure and catalytic performance (Figure 16c) [193]. By carefully adjusting parameters such as etching time, temperature, and solution concentration, researchers can precisely control the density of S-SA vacancies. Both experimental and theoretical analyses reveal that uniformly distributed S-SA vacancies in MoS_2_ catalysts outperform clustered vacancies in terms of HER performance. The absence of substrate atoms due to vacancies creates “sites” for supported atoms, preventing their aggregation (Figure 16d) [193]. The size of these “sites” influences the size and coordination structure of supported atoms. Moreover, from a charge perspective, missing atoms are typically replaced by atoms with similar charges or zero-valence atoms to maintain charge balance. For instance, cation defects are usually filled by metal atoms, while anion defects are filled by non-metal atoms. Furthermore, anion defects disperse metal atoms and cation defects alter non-metal atoms. This defect modification further fine-tunes the electronic structure, optimizing adsorption energy and enhancing electrocatalytic activity [194,195].

### 4.4. Charge Transfer

**Figure 17:**
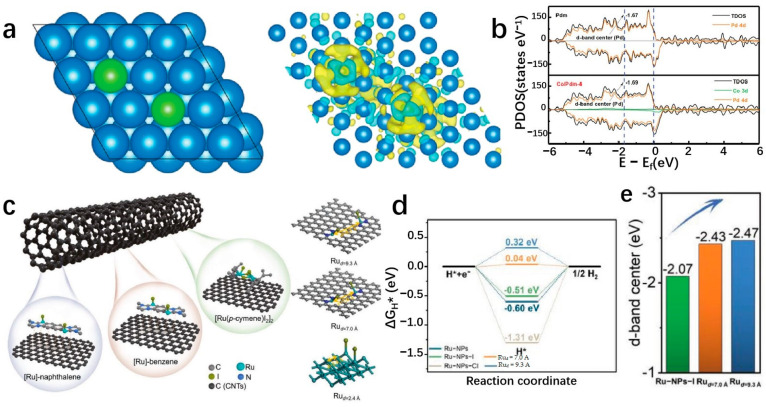
(**a**) Top view of the slab model of Co/PDM-4 [197]. (**b**) DOS for PDM and Co/PDM-4 [197]. Copyright 2023, Wiley-VCH. (**c**) Schematic diagram of the designed catalysts [47]. (**d**) Gibbs free energy diagram for H adsorbed on Ru [47]. (**e**) d-band center for Ru [47]. Copyright 2023, Wiley-VCH.

The chemical bonds between SAs and their supports are typically established through shared electron pairs or electron transfer, resulting in enhanced connectivity and stability [196]. These bonds often exhibit clear directionality, which aids in determining the molecular structure and properties. Yang et al. designed the engineered form of Pd, fixed a single Co atom on the Pd metalene (PDM) support, obtained Co/PDM, and realized the preparation of ultra-thin 2D SAC. The unsaturated coordination environment, combined with the unique geometric and electronic structure, enables the modulation and charge redistribution of the d-band center, resulting in highly active electronic states on the Co/PDM surface (Figure 17a) [197]. Doping of Co atoms can change the electronic structure and charge distribution on the PDM surface, further optimizing the Fermi level and the electronic state near the center of the d band, which allows the single atoms on the PDM surface to be tightly fixed to the surface (Figure 17b) [197]. First-principles studies demonstrate that charge transfer plays a key role in modulating the d-orbital electron occupancy of single metal atoms, thereby enhancing catalytic activity for HER. New 2D MBenes can be synthesized by selectively etching the intermediate Al layer in the parent MAB phase. Unlike carbon in MXenes, boron in MBenes, with lower electronegativity, creates a unique electronic state for Pt-B interactions, enabling Pt-SAs to reach a zero-valent state and exhibit catalytic behavior similar to metallic Pt surfaces. In this study, Pt-SAs are integrated into the MBenes structure through substitutional doping at Mo sites and adsorbed onto the MBenes structure via favorable Pt-B or Pt-Mo interactions, increasing the number of electrons in the 5d orbitals and enhancing catalytic activity for the HER. SACs with specific metal–metal interactions at designated sites outperform traditional catalysts with isolated metal sites. Long-range electronic interactions between single atoms (SAs) significantly impact catalytic performance, which is crucial for understanding and improving the reaction kinetics of metal site-controlled catalysis. A study utilized planar organometallic molecular design to precisely control the spacing between SAs (Figure 17c) [47], creating three types of Ru c-SACs with Ru-SA atomic distances (SAD) ranging from 2.4 to 9.3 Å. Tuning the Ru SAD to 7.0 Å led to an exceptionally high turnover frequency of 17.92 H_2_ s^−1^ and a remarkable mass activity of 100.4 A mg^−1^ at overpotentials of 50 and 100 mV, respectively, surpassing previous Ru-based catalysts. Additionally (Figure 17d) [47], DFT calculations confirmed that the negative correlation between Ru SAD and the d-band center, influenced by long-range interactions and local atomic geometries, results in an optimal electrostatic potential and the highest catalytic activity at a Ru atomic distance of 7.0 Å. In SAC systems, anchoring mechanisms include covalent bonds with coordinating atoms, anchoring at vacancy sites, or electron transfer through long-range interactions in the second coordination shell. Efficient electron transfer regulation adjusts the electronic structure and d-band center of SA sites, offering a variety of methods to fine-tune the electronic structure of SAs (Figure 17e) [47].

### 4.5. Confined Environment Structure Design

**Figure 18:**
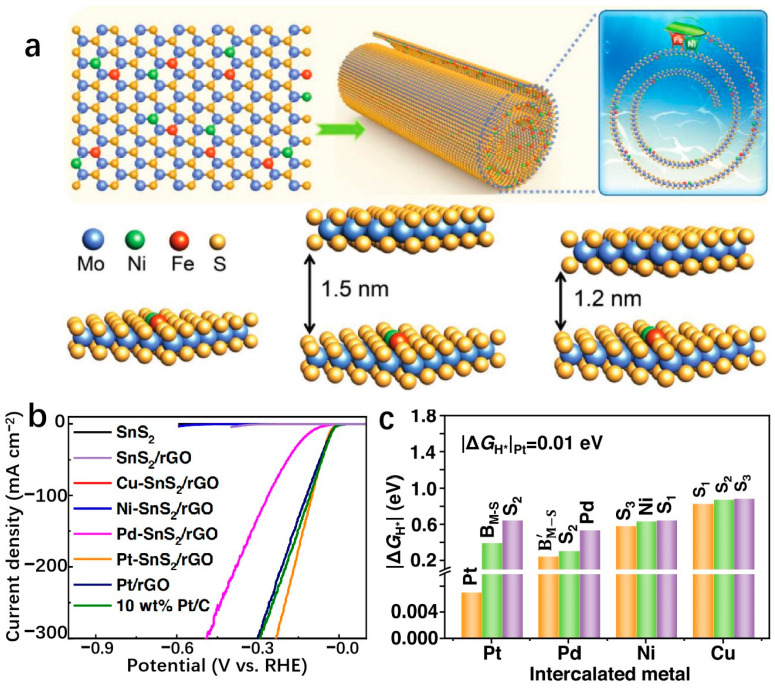
(**a**) The plane NiFe@MoS_2_ is interlayer constrained [210]. Copyright 2023, Wiley-VCH. (**b**) LSV curves of samples [211]. (**c**) The smallest three ΔG_H*_ values of Pt, Pd, Ni, and Cu-SnS_2_, respectively [211]. Copyright 2022, Springer Nature.

Confined structure design encompasses micropore region confinement [198,199,200,201,202], lattice confinement [203,204,205], defect confinement [206], and more. Spatial confinement strategies have been proven effective in creating well-defined SA catalyst microenvironments. In the micropore region, SAs are confined or encapsulated within nano or sub-nano-scale spaces [207]. In these microenvironments, interactions between reactants and catalysts are spatially restricted, leading to more controllable reaction pathways and intermediates, thereby enhancing reaction specificity and product yield [208,209]. Lattice-confined and defect-confined SA sites can effectively improve metal–support interactions, while also increasing immobilization and preventing SA aggregation. As shown in Figure 18a [210], by simultaneously anchoring Ni and Fe-SA within the interlayer of MoS_2_ at the 2D scale, this configuration not only preserves the high activity advantages of the dual SA species but also provides the confined catalyst with enhanced adsorption equilibrium, intrinsic activity, and electrical conductivity. Additionally, the interlayer confined structure significantly enhances the stability of the catalyst, resulting in an efficient and stable low-cost electrocatalyst for overall water splitting in acidic conditions. Theoretical calculations and experimental results confirm that the confined structure plays a critical role in promoting efficient water splitting and safeguarding the dual SA from harsh acidic environments in this dual-functional NiFe-SA catalyst. Another strategy involves intercalating various metal atoms within the layers of SnS_2_ to form SACs (Figure 18b) [211]. This configuration of SACs demonstrates high electrocatalytic activity and stability. Theoretical calculations suggest that intercalated metals activate the 2D material, transforming the previously inert material into a reactive substance. In comparison to conventional SACs, SACs confined within van der Waals gaps can maintain a coordination-unsaturated state without direct exposure to the catalytic environment, potentially offering enhanced stability. The interlayer regions of the 2D support, created through van der Waals interactions, establish an ideal confined space that acts as a stable nanoscale site for single atoms. This confined interlayer space facilitates the movement of intermediates, reactants, and products within the confined area, enabling the catalyst to achieve high activity, selectivity, and stability. It provides an innovative support structure for the catalytic performance of SA active sites. Lattice confinement stabilizes single-atom species through strong bonding, ensuring the long-term stability of SAs. For example, by employing a lattice confinement method, Ru nanoparticles (RuNP) are combined with Ru single atoms and embedded into a crystalline fullerene network (CFN) (RuNP-Ru_SA_@CFN-800) (Figure 18c) [212]. The robust CFN matrix and lattice confinement approach guarantees the catalyst’s exceptional long-term stability, achieving stability for over 1400 h at an industrial scale. The design of confined structures presents a new approach to achieving advancements in the performance and stability of single-atom catalysts supported on 2D materials.

### 4.6. Other Methods for Manipulating the Microenvironment of Single Atoms

**Figure 19:**
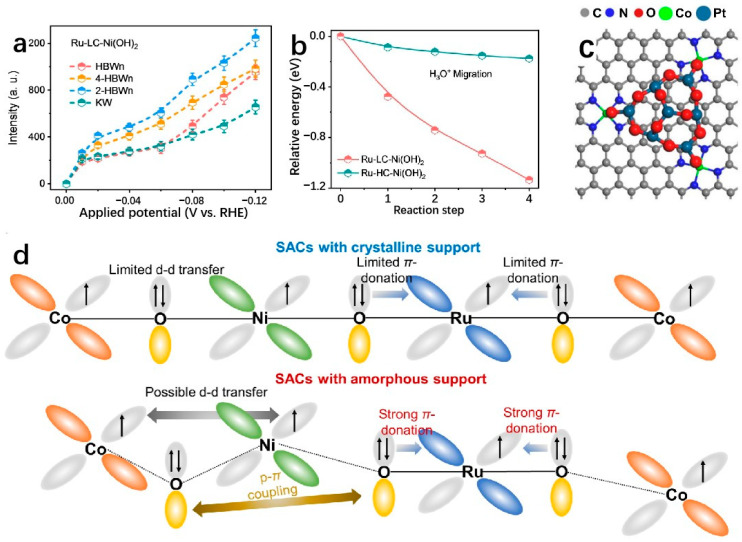
(**a**) Intensity changes in characteristic peaks during the structural evolution of water at the Ru-LC-Ni(OH)_2_ interface. And energy barriers for proton migration on Ru-LC-Ni(OH)_2_ and Ru-HC-Ni(OH)_2_ [213]. (**b**) Density functional optimization of amplified Pt-ACs/CoNC structures [213]. Copyright 2023, Wiley-VCH. (**c**) Schematic representations of the electronic coupling among Co-O-Ni-O-Ru-O-Co unit in amorphous and crystalline SACs [214]. Copyright 2022, Springer Nature. (**d**) Electron coupling diagram of Co-O-Ni-O-Ru-O-Co unit in amorphous and crystalline SACs [215]. Copyright 2021, Wiley-VCH.

In SACs, the MSI is primarily influenced by the local coordination environment of the support, which is determined by the electronic exchange between the SA and support. The MSI induced by SAs on amorphous versus crystalline support surfaces is significantly different, leading to distinct structure–activity relationships in the HER process. Amorphous supports, in contrast to crystalline supports, possess numerous metal dangling bonds with unsaturated electronic configurations. This results in more frequent orbital coupling that redistributes local lone pair electrons and accelerates charge transfer between active sites. Therefore, comprehending the unique MSI between amorphous support and external SA is essential for improving the electrochemical activity and stability of SACs. For instance, in a study involving Ru-SA supported on low-crystalline nickel hydroxide (Ru-LC-Ni(OH)_2_) (Figure 19a) [213], the low oxidation state Ru-SA enhances the adsorption of hydronium ions (H_3_O^+^), creating a local acidic microenvironment in alkaline media that boosts the HER activity. Conversely, the Ru-SA catalyst supported on Ru-HC-Ni(OH)_2_ demonstrates slower catalytic reaction rates. In surface-supported SACs, individual atoms are widely spaced apart, lacking interaction. Conversely, atom clusters (ACs) contain neighboring metal atoms with active sites, each atom maintaining independence. ACs, while achieving a high atomic utilization rate, improve catalytic performance through synergistic effects among adjacent atoms. However, atom clusters are prone to aggregation into nanoparticles. To address this, some researchers have developed an N-doped porous carbon support with uniformly dispersed Co atoms, acting as anchors for Pt species, Co atoms create a strong metal–support interaction that effectively prevents Pt-ACs from aggregating into nanoparticles. The distinct properties of Pt-O-Pt units within Pt-ACs facilitate water dissociation and H* adsorption. Heterostructure supports with 2D structures and porous characteristics enhance the loading and exposure surface of SAs and active sites (Figure 19b) [213]. The development of simple, environmentally friendly, and efficient SA dispersed heterostructure nanosheets holds significant importance for the commercial application of SACs. Theoretical calculations exploring various phase interfaces and atomic coordination environments of SAs suggest that the heterostructure interface optimizes the electronic structure and H* adsorption energy of the entire catalyst, effectively enhancing the electron transfer ability from active sites to water molecules (Figure 19c) [214]. In many 2D support-based SAs, the monatomic structures loaded on the support surface regulate the SA sites through multiple electronic interactions. Figure 19d [215] illustrates the structure of single-atoms ruthenium (Ru-SAs) supported on amorphous cobalt/nickel hydroxide (oxide) (Ru-a-CoNi), which features a 2D flake-like stacked and intertwined structure with randomly distributed Ru-SAs. The amorphous configuration induces d-d electron transfer and medium to long-range p-π orbital coupling, enhancing the MSI. This configuration promotes electron exchange between Ru-SAs in the local Ru-O-Co/Ni configuration through strong π donation to the electron-deficient t_2_g d orbitals of Ru^δ+^, facilitating effective π-symmetric electron transfer and creating a tighter MSI between a-CoNi and Ru. The resulting configuration demonstrates outstanding durability and HER activity. The shorter bond length between Ru-SAs and the amorphous framework indicates a tighter MSI, assisting in electron redistribution within the Ru-a-Co/Ni local configuration. Moreover, the presence of defect sites and unpaired electrons in the amorphous state not only distorts the Ru-O-Co/Ni local bonds, impacting electron redistribution and orbital coupling but also exposes active sites to reaction intermediates, thereby increasing the electrochemical active area of Ru-a-CoNi. Two-dimensional support materials have attracted the interest of researchers because of their distinct surface effects and consistently exposed surfaces. The various forms of interaction between SA sites and 2D supports offer a broader scope for mechanistic studies. The advancement of 2D support materials and the investigation into the interaction mechanisms between SA sites and 2D materials are anticipated to facilitate the practical use of SACs.

## 5. Conclusions and Outlook

### 5.1. Conclusions

In recent years, SACs supported on 2D materials have been the subject of extensive research. Various surface morphologies and electronic structures of the supporting surfaces have been investigated, resulting in the proposal of numerous reaction mechanisms. As a result, SACs supported on 2D materials have shown distinct advantages. Furthermore, the growing diversity in synthesis methods for 2D materials and the loading of SAs has led to a significant increase in SACs supported on 2D materials. This paper provides a comprehensive review of the historical development of SACs supported on 2D materials, covering synthesis methods, characterization techniques, interaction mechanisms, and the mechanisms involved in the HER.

Carbon supports are the most widely used carrier materials for electrocatalysis due to their good conductivity, large specific surface area, porous surface structure, and low material cost. Carbon supports contain rich functional groups and defects, which can greatly load more SAs and adjust the anchor point through defects. However, it is difficult to avoid the poor stability of SACs supported by carbon materials. In contrast, 2D nanomaterials have larger specific surface areas, suitable layer spacing, high selectivity, and good stability, which may endow SACs with good electrocatalytic water splitting performances via strong interaction between SAs and supports.

(1) The article provides an overview of common methods used in the synthesis of 2D support materials and discusses various strategies for loading SAs onto these supports. The goal is to establish a connection between the synthesis conditions of 2D materials and the interactions between SAs and the supports. However, there is still a need for precise control over the electronic states of SA sites within the microenvironment of 2D support surfaces. The development of synthesis conditions and optimization strategies is essential for creating the desired microenvironment on the surfaces of 2D materials. Ultimately, the aim is to develop synthesis methods for 2D materials and loading strategies for SAs that enable low-cost, large-scale synthesis of high-performance SACs.

(2) A summary of commonly used characterization techniques for SAs is provided, along with an analysis of the characteristics and advantages of various methods in the characterization of SACs. While these techniques may not fully uncover the interaction between single atoms and 2D support materials, the combined use of multiple advanced characterization techniques and theoretical calculations is crucial for gaining insights into the structure-performance relationship between single atoms and 2D support.

(3) This paper presents a comprehensive overview of electronic structure construction methods and interaction mechanisms on the surfaces of 2D support in the field of HER, drawing on preparation strategies for SACs and advanced characterization methods. It discusses the impact of atomic doping, defect engineering, electron transfer, and trap structure on the surfaces of 2D support in optimizing SAC performance. Theoretical advancements in d-band center modulation, charge distribution, and molecular orbital theory are also examined. Building on this theoretical foundation, the paper delves into the structural design and structure-performance relationship of SACs supported by 2D materials, emphasizing trends in structural innovation, interface regulation, and mechanistic studies.

### 5.2. Outlook

Water electrolyzers have attracted wide research interest because of their fast time response, high current density, and high hydrogen purity. However, the cathodes and anodes often require noble metal catalysts, while the high price of noble metals is one of the key factors to prevent large-scale application. SACs with the highest atomic utilization efficiency can reduce the loading amount of noble metals. Unfortunately, the durability of SACs remains a challenge under the operating conditions of water electrolyzers. Therefore, it is essential to develop a stable, efficient, and low-cost SAC for use in electrolyzers.

Supported by 2D support, SACs have shown significant research advancements, but there are still lingering issues. Future development directions in this area include:

(1) The current range of 2D support materials is limited, with commonly used materials including graphene and carbon nitride. However, there is a need for thorough exploration of other 2D carbon-based materials such as amorphous carbon, 2D MOF-derived carbon, and 2D COF-derived carbon. Most studies on metal single-atom active sites have focused on transition metal elements like Ni, Co, Fe, Ru, Pt, and Pd, with dopants primarily limited to these transition metals. Research on lanthanides, actinides, and other metal elements is lacking, and non-metal elements are rarely considered. Furthermore, there is a need for extensive research on new species in non-carbon materials like MXenes, MBenes, 2D COF materials, and 2D MOF materials. These materials offer a wider variety of surface functional groups, diverse structures, and abundant pore structures, which provide significant advantages for anchoring and confining SA species.

(2) Current SAC preparation methods are complex, resulting in significant waste generation and high production costs, which hinders commercialization. Therefore, there is a crucial need to develop innovative, simple, and efficient preparation techniques for SACs, using cost-effective support materials and straightforward synthesis approaches to minimize waste. Additionally, exploring scalable production methods is essential to accelerate the commercialization of SACs.

(3) The stability of SAs in catalysis poses a persistent challenge, primarily due to their high surface free energy leading to poor stability in SACs. Several strategies have been suggested to tackle this issue, such as anchoring SAs using vacancies, doping with atoms that create strong bonds, and employing organic functional groups for anchoring. Despite these efforts, achieving a stability level suitable for industrial applications remains difficult. A promising approach to enhance the stability of SAs on support surfaces involves implementing multiple reinforcement mechanisms simultaneously. This could involve doping porous surfaces with elements that form strong bonds, attaching organic groups to the single atoms, and combining physical isolation with various chemical fixation methods to stabilize the SAs.

(4) The aggregation of single atoms is one of the most emerging challenges for SACs, which often results in low load capacity of SAs. To address this, there is an urgent need to explore efficient synthetic methods for high dispersion and precise adjustment of the anchoring position of the support surface for high load amounts of SAs.

(5) The restrained activity of each single atom obstructs the performance of SACs. To overcome this drawback, maximizing the inherent activity of each isolated site is an effective method to improve the overall electrocatalytic performance. Atomically dispersing another metallic element into the structure to form bimetallic sites may become a promising strategy.

## Figures and Tables

**Table 1:** HER properties and synthesis strategies of representative monatomic catalysts.

Catalyst	Electrolyte	Overpotential (mV)	Current Density (mA cm^−2^)	Tafel Slope (mV dec^−1^)	Strategy	Ref.
Co-CNG	1 M KOH	47	10	44	Annealing process	[106]
Pt@DG	0.5 M H_2_SO_4_	30	10	53	Annealing process	[107]
	1 M KOH	37	10	-		
Pt_SA_Co(OH)_2_@Ag	1 M KOH	29	10	35.72	Electrochemical phase transformation	[116]
Ru_1_/D-NiFe LDH	1 M KOH	18	10	29	Electrochemical deposition	[117]
Pt-Ni(OH)_2_-E	1 M KOH	21	10	51	Electrochemical deposition	[118]
	1 M PBS	34	10	-		
NiIr_SAA_-NiFe-LDH	1 M KOH	28.5	10	32.9	Hydrothermal method	[120]
s-Pt/1T′-MoS_2_	0.5 M H_2_SO_4_	50	10	10	Electrochemical intercalation	[130]
Cu_0.39_MoS_2_	0.5 M H_2_SO_4_	250	10	52	Hydrothermal synthesis	[72]
SAP-Mo_2_C-CS	0.5 M H_2_SO_4_	36	10	38.1	Pressurized gas-assisted process	[132]
Ru_SA_-N-Ti_3_C_2_T*_x_*	1 M KOH	27	10	29	Acoustic wave assisted liquid phase stripping	[138]
	0.5 M H_2_SO_4_	23	10	42		
	1 M PBS	81	10	-		
Ru_SA_@Ti_3_C_2_T_x_	1 M KOH	425.7	1000	-	Wet chemistry impregnation	[139]
Ru_SA_-N-S-Ti_3_C_2_T_x_	0.5 M H_2_SO_4_	76	10	90	Annealing process	[140]
Ti_3_C_2_T_x_-Pt_SA_	0.5 M H_2_SO_4_	38	10	45	Acoustic wave assisted liquid phase stripping	[84]
MXene-Fe-MOF	0.5 M H_2_SO_4_	52	10	~30	Selective etching-assisted liquid phase exfoliation.	[143]
h-BN	1 M KOH	28	10	25	Annealing method	[152]
NGA-COF@Pt	NGA-COF@Pt	13	10	21.88	Electrochemical modification strategy	[158]

## Data Availability

No new data were created or analyzed in this study. Data sharing is not applicable to this article.
